# Analysis of Physiological and Transcriptomic Differences between a Premature Senescence Mutant (*GSm*) and Its Wild-Type in Common Wheat (*Triticum aestivum* L.)

**DOI:** 10.3390/biology11060904

**Published:** 2022-06-12

**Authors:** Juan Lu, Lili Sun, Xiujuan Jin, Md Ashraful Islam, Feng Guo, Xiaosha Tang, Kai Zhao, Huifang Hao, Ning Li, Wenjun Zhang, Yugang Shi, Shuguang Wang, Daizhen Sun

**Affiliations:** State Key Laboratory of Sustainable Dryland Agriculture, College of Agriculture, Shanxi Agricultural University, Jinzhong 030801, China; lujuannxy@163.com (J.L.); sunlili0818@163.com (L.S.); 15582408175@163.com (X.J.); a.islam160@sxau.edu.cn (M.A.I.); gf980403@163.com (F.G.); tangxiaosha123@126.com (X.T.); zk51712@163.com (K.Z.); jyhf2009@163.com (H.H.); 13159862006@163.com (N.L.); zhangwenjun9876@163.com (W.Z.); shiyugang0804@126.com (Y.S.); wsg6162@126.com (S.W.)

**Keywords:** common wheat, differentially expressed genes, leaf senescence, premature senescence mutant, RNA sequencing

## Abstract

**Simple Summary:**

Early leaf senescence is an important agronomic trait that affects crop yield and quality. To understand the molecular mechanism of early leaf senescence, a wheat (*Triticum aestivum* L.) premature leaf senescence mutant (*GSm*) and its wild type were employed in this study. We compared the physiological characteristics and transcriptome of wheat leaves between the wild type (WT) and the mutant at two-time points. Physiological characteristics and differentially expressed gene (DEG) analysis revealed many genes and metabolic pathways that were closely related to senescence. These results will not only support further gene cloning and functional analysis of *GSm*, but also facilitate the study of leaf senescence in wheat.

**Abstract:**

Premature leaf senescence has a profound influence on crop yield and quality. Here, a stable premature senescence mutant (*GSm*) was obtained from the common wheat (*Triticum aestivum* L.) cultivar Chang 6878 by mutagenesis with ethyl methanesulfonate. The differences between the *GSm* mutant and its wild-type (WT) were analyzed in terms of yield characteristics, photosynthetic fluorescence indices, and senescence-related physiological parameters. RNA sequencing was used to reveal gene expression differences between *GSm* and WT. The results showed that the yield of *GSm* was considerably lower than that of WT. The net photosynthetic rate, transpiration rate, maximum quantum yield, non-photochemical quenching coefficient, photosynthetic electron transport rate, soluble protein, peroxidase activity, and catalase activity all remarkably decreased in flag leaves of *GSm*, whereas malondialdehyde content distinctively increased compared with those of WT. The analysis of differentially expressed genes indicated blockade of chlorophyll and carotenoid biosynthesis, accelerated degradation of chlorophyll, and diminished photosynthetic capacity in mutant leaves; brassinolide might facilitate chlorophyll breakdown and consequently accelerate leaf senescence. *NAC* genes positively regulated the senescence process. Compared with *NAC* genes, expression of *WRKY* and *MYB* genes was induced earlier in the mutant possibly due to increased levels of reactive oxygen species and plant hormones (e.g., brassinolide, salicylic acid, and jasmonic acid), thereby accelerating leaf senescence. Furthermore, the antioxidant system played a role in minimizing oxidative damage in the mutant. These results provides novel insight into the molecular mechanisms of premature leaf senescence in crops.

## 1. Introduction

Common wheat (*Triticum aestivum* L.) is one of the world’s major cereal crops and it provides ~20% of global calorie consumption (https://www.fao.org/faostat/en/#data accessed on 29 November 2021). The global wheat consumption has grown to ~700 million tons year^–1^ over the past four years [1]. Grain yield of common wheat depends primarily on the accumulation of photosynthetic products in functional leaves during the grain-filling stage. Therefore, prolonging leaf stay-green and photosynthesis duration is pivotal to achieve high wheat yield [2].

Plant premature senescence refers to the phenomenon that plants undergo physiological decline in advance in the normal functional period, which is usually caused by external environmental stress or self-gene mutation [3,4]. Usually accompanied by the change of leaf color, the stability of photosynthetic pigments decreases, the photosynthetic rate decreases, the transportation capacity of leaves decreases, the content and activity of various reactive oxygen species scavengers decrease, and eventually the plants die prematurely [5]. The leaves are a vital organ for crop photosynthesis, so that premature leaf senescence not only determines crop yield but also influences crop quality [6,7]. Progression of senescence is followed by an increase in the degradation of RNA, protein, lipid and DNA degradation to be recycled into the sink organ [8]. About 80% of nitrogen contained in the grain is provided by the salvaging of chloroplast proteins during the senescence process [9]. Delaying senescence and increasing the length of photosynthetic activity have been reported to increase grain yield, while increasing the rate of amino acid remobilization to degrade proteins in accelerated senescence can increase the grain protein content [10]. Understanding the cause of premature leaf senescence is crucial to elucidate the molecular mechanisms of plant senescence and source/sink relations.

Many premature senescence mutants have been found in crops, yet the causes of premature senescence are variable. The most common type of plant premature senescence arises from mutations in genes related to chlorophyll metabolism. For example, elevations in *NYC1*, *SGR*, *PPH*, and *PAO* gene expression levels lead to earlier leaf senescence in *Arabidopsis thaliana* [11]. Additionally, gene silencing of *CHLI* and *CHLD* results in a premature senescence mutant in pea (*Pisum sativum* L), which exhibits a yellow leaf phenotype and diminished magnesium chelatase activity [12]. Owing to *CHLI* gene mutation, the plant leaves of a tobacco (*Nicotiana tabacum* L.) premature senescence mutant present a golden yellow color [13]. Furthermore, a corn (*Zea mays* L.) yellow-green leaf mutant, *ygl-1*, shows premature senescence because of decreased chlorophyll and carotenoid content [14].

The second type of plant premature senescence is attributed to gene mutations induced by abnormal development of chloroplasts. An example is barley (*Hordeum vulgare* L.) plastome mutant, *cytoplasmic line 2* (*CL2*), in which the seedling phenotype is changed from light green to albino color at emergence as a result of abnormal chloroplast development [15]. Moreover, the *mgd1* mutant of *A. thaliana* shows premature senescence due to mutation in the *MGD1* gene of lipid monogalactosyldiacylglycerol (MGD) synthase, which catalyzes biosynthesis of MGD, a major structural component of the photosynthetic membrane in chloroplasts [16]. In another *A. thaliana* mutant, *pca*, both the cotyledons and leaves are light-yellow or white in color owing to abnormal chloroplast development [17].

The third type of plant premature senescence is triggered by mutations in protease genes. For example, a transposon insertion leads to alternative splicing errors in two *pre2* alleles (encoding phytochrome-dependent late-flowering protein PHL) of the corn mutant *pre2*, causing deletion of multiple glutamines near the C-terminus of mature transcripts and consequently premature senescence of plants [18]. A single nucleotide deletion in the open reading frame of pectate lyase contributes to premature leaf senescence in the *ospse1* mutant of rice (*Oryza sativa* L.) [19]. Because the pho2 protein encoded by *pho2* is abnormal in the *A. thaliana* mutant *pho2*, substantial accumulation of phosphorus occurs in the shoots, resulting a spotted and yellowing phenotype [20].

The fourth type of plant premature senescence is due to mutations in genes related to hormone equilibrium. For example, exogenous application of jasmonic acid (JA) leads to earlier senescence in attached and detached leaves of wild-type (WT) *A. thaliana*, but fails to induce premature senescence in plants of the JA-insensitive mutant *coi1*; this suggests that JA signaling is required for JA to facilitate leaf senescence [21]. Endogenous levels of abscisic acid (ABA) in the rice premature senescence mutant *psl85* increase considerably in the late stage of aging, indicating that *psl85* takes part in ABA-induced leaf senescence [22]. Compared with WT, the tobacco leaf senescence mutant *yl1* shows higher mones [23]. An auxin-auxotrophic mutant of tobacco, *IVA3*, shows premature death [24].

The fifth type of plant premature senescence is caused by transcription factor mutations. Some senescence-related transcription factors are reported to participate in the regulation of plant hormone levels and then influence leaf senescence. For example, a novel WRKY-type leaf senescence promoter, *TaWRKY42-B*, has been identified in common wheat. *TaWRKY42-B* interacts with *AtLOX3* and its orthologous *TaLOX* (*TraesCS4B02G295200*) to facilitate JA biosynthesis and thereby accelerate initiation of leaf senescence [25]. *WRKY57* plays a major role in JA-induced leaf senescence [26], while *WRKY45* regulates leaf senescence by modulating GA signaling pathway [27]. *ABF2/3/4* and *ABI5* accelerate chlorophyll degradation and leaf senescence through promoting ABA biosynthesis [28]. Plant premature senescence can also be caused by environmental induction [29,30], reactive oxygen species (ROS) damage [31], carbohydrate biosynthesis blockade [32], and energy metabolism blockade [33,34].

In recent years, along with the development of high-throughput sequencing technology, transcriptome sequencing (RNA-seq) has emerged as a powerful tool to study complex biological processes at the molecular level and identify candidate genes involved in specific biological functions. There is no exception for premature senescence in plants, such as the mechanisms of leaf color formation in an anthurium (*Anthurium andraeanum* Linden cv. ‘Sonate’) mutant [35], the mechanisms of yellow leaf mutation in crape myrtle (*Lagerstroemia indica* L.) [36], and the gene regulatory networks of premature leaf senescence in corn [37], rice [38], and wheat [39,40].

In an earlier study, we used ethyl methane sulfonate (EMS) to treat the common wheat cultivar Chang 6878 and found a premature-senescence homozygous mutant in M3. Senescence was initiated in first leaf of the mutant in the three-leaf stage; subsequently, in the three-leaf-one-leaflet stage, the first leaf turned yellow except for its leaf base, whereas the second leaf started yellowing and senescence at its leaf tip, showing an overall trend of growing with senescence. Accordingly, the mutant was designated *Growing with Senescence mutant* (*GSm*). To understand the cause of premature senescence in the mutant, we first measured associated physiological parameters in *GSm* and WT. Then, we carried out RNA-seq analysis of flag leaves in *GSm* and WT for two stages after leaf full expansion (2 d, S1; 9 d, S2). The aim of this study was to unravel the cause of premature senescence in the *GSm* mutant at the transcriptomic level.

## 2. Materials and Methods

### 2.1. Experimental Material Preparation and Agronomic Analysis

In 2013, we used EMS to mutagenize the common wheat cultivar Chang 6878. The mutagenized material was continuously planted in the experimental base of the Wheat Research Institute, Shanxi Academy of Agricultural Sciences (Linfen, Shanxi Province, China) during 2013–2017. A mutant with a stable mutant phenotype, designated *GSm*, was formed after multiple generations of self-crossing. From 2017 to 2021, the mutant *GSm* and WT were planted in the wheat experimental field (37°25′ N, 112°25′ E) in the College of Agriculture, Shanxi Agricultural University (Jinzhong, Shanxi Province, China). During this period, the obtained genetically stable homozygous *GSm* and WT were used to observe their phenotypic differences throughout the whole growth period in 2019. Ten mutant and WT plants with uniform growth were selected at random after maturity to investigate agronomic traits such as plant height, thousand-grain weight, and seed setting rate. The mean trait values of the 10 selected plants were used for data analysis.

### 2.2. Measurement of Photosynthetic Performance

*GSm* and WT plants (nine each) with uniform growth were selected at random after wheat flag leaves were fully unfolded. The plants were marked for subsequent measurements of chlorophyll content and photosynthetic fluorescence characteristics from the day of leaf full expansion to maturity at 3 d intervals. The relative chlorophyll content (SPAD value) was measured on flag leaves using a chlorophyll meter (SPAD-502; Minolta, Osaka, Japan). Pn and Tr were measured at 9:00–11:00 using a handheld photosynthesis system (CI-340; CID, Beijing, China). Fv/Fm, NPQ, and ETR were measured in the middle part of the leaf using a portable modulated chlorophyll fluorometer (MINI-PAM; Walz, Effeltrich, Germany) after dark treatment (20 min) with a dark treatment clip.

### 2.3. Analysis of Senescence-Related Physiological Parameters

After full expansion of wheat flag leaves, *GSm* and WT plants (50 each) with uniform growth were selected at random and marked for subsequent sampling. Flag leaf of main spike were sampled at 3 d intervals from the day of full leaf expansion to maturity. MDA content was analyzed with 5% thiobarbituric acid [41,42], and soluble protein content was determined using Coomassie brilliant blue [43]. CAT activity was assayed by UV absorption spectroscopy [44] and POD activity was determined with guaiacol as the substrate [45].

### 2.4. RNA Extraction, Library Construction, and Sequencing

WT and *GSm* seeds were sown in Shanxi (China) on 4 October 2020. The flag leaves of WT and *GSm* were collected at 2 d after full expansion of flag leaves, denoted as WS1 and MS1, respectively. The flag leaves of WT and *GSm* were collected again at 9 d after full expansion of flag leaves, denoted as WS2 and MS2, respectively. A total of 12 samples from three biological replicates of WT and *GSm* were used to construct cDNA libraries. All samples were immediately frozen in liquid nitrogen and stored at −80 °C until RNA extraction. Total RNA was extracted using Trizol reagent (Invitrogen Life Technologies, Shanghai, China) and then treated with RNase-free DNase I (TaKaRa, Dalian, China). RNA purity and integrity were checked prior to cDNA library construction. The cDNA library construction and Illumina sequencing of the 12 RNA samples were completed by Shanghai Majorbio Co., Ltd. (Shanghai, China).

### 2.5. Bioinformatics Analysis of RNA-Seq Data

The raw reads were cleaned by removing adapter and low-quality sequences. The clean reads were mapped to a cDNA database from the wheat variety Chinese Spring (IWGSC_RefSeq_v1.1). The gene length and sequencing depth were normalized by TPM (Transcripts Per Million reads). Differences in RNA transcript levels were analyzed using DESeq2 and edgeR. p-adjusted < 0.05 and |log2(fold-change)| ≥ 1 were set as the threshold for identifying DEGs. GO enrichment analysis of DEGs was performed using the GO database (http://www.geneontology.org accessed on 16 April 2022). KEGG pathway enrichment analysis was performed on DEGs using the KEGG database (http://www.genome.jp/kegg/ accessed on 16 April 2022). All raw reading sequences were uploaded in NCBI’s sequence read archive (SRA) under the accession number PRJNA823852.

### 2.6. Quantitative Reverse-Transcription PCR

To verify the accuracy of the transcriptome data, we randomly selected multiple DEGs for expression analysis by qRT-PCR. According to the instructions of RNAiso Plus (9108) extraction reagent (TaKaRa), the total RNA of leaves of different periods and varieties was extracted. Select RNA of good quality extracted as template, and perform reverse transcription according to the instructions of PrimeScript™ RT reagent Kit with gDNA Eraser (Perfect Real Time) (RR047) reverse transcription kit (TaKaRa). qRT-PCR was performed in a 96-well fluorescence quantitative PCR machine CFX96 (BIO-RAD, Hercules, CA, USA) according to the instructions of TB Green^®^ Premix Ex Taq ™ II (Tli RNaseH Plus) (RR820) fluorescence quantitative kit (TaKaRa). The reaction system was 10 μL of TB Green Premix Ex Taq II, 0.4 μL of ROX, 0.4 μL each of pre- and post-quantitative primers (10 μM), 7.8 μL of ddH2O, and 1 μL of template cDNA. The reaction program was: 95 °C, 3 min; 95 °C, 20 s, 60 °C, 20 s, 72 °C, 20 s, 40 cycles, the fluorescence signal was collected after each cycle, and then entered the melting curve stage, 60 °C, 5 s, start at 65 °C, increase to 95 °C in steps of 0.5 °C, and hold each temperature for 5 s. Supplementary Table S7 lists the specific primer sequences, with Actin as the internal reference.

## 3. Results

### 3.1. Manifestations of the Premature Senescence Mutant (GSm)

**Figure 1:**
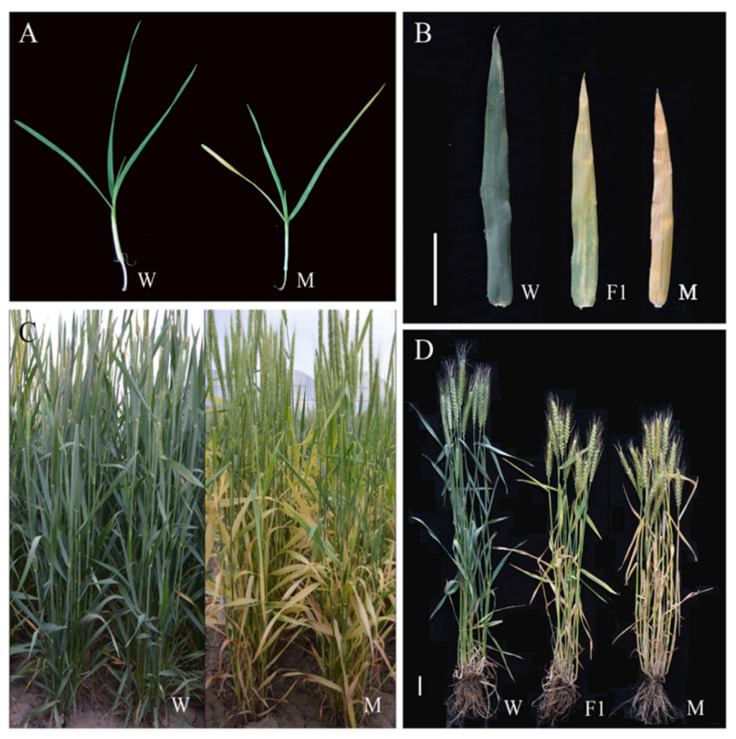
Phenotypes of wild-type (Chang6878) and mutant (*GSm*) wheat at (**A**) seedling of Chang6878, and *GSm* (**B**) mid-grouting stage (flag leaf) of Chang6878, hybrid F1 and *GSm*, (**C**) flowering stage of Chang6878, and *GSm*, and (**D**) mid-grouting stage of Chang6878, hybrid F1, and *GSm*.

The *GSm* mutant was acquired from the common wheat cultivar Chang 6878 by EMS mutagenesis. Leaf yellowing and senescence were initiated at the tip of the first leaf in *GSm* during the three-leaf stage. Then, the first leaf turned yellow in most part of it (except for leaf base), while yellowing and senescence were initiated at the tip of the second leaf during the three-leaf-one-leaflet stage. After that, the first leaf turned completely yellow, the second leaf also turned yellow in most part of it (except for leaf base), while senescence was initiated at the tip of the third leaf in the four-leaf stage. The leaf senescence occurred in a progressive manner. Along with the growth of the plants, completely fully unfolded leaves started yellowing after the jointing stage. The yellowing was initiated in flag leaves from the heading stage, and all leaves turned yellowish green in the early grain-filling stage. Compared with WT, F1 plants had no difference in the seedling stage and their basal leaves gradually turned yellow from the jointing stage. The rate of senescence in F1 plants was distinctively lower than that of the *GSm* mutant, yet greater than that of WT (Figure 1).

Then, we measured plant agronomic traits in the *GSm* mutant and WT. Except for number of sterile spikelets, flag leaf length, and penultimate leaf length, all other agronomic trait values significantly decreased in *GSm* than in WT. Among these, yield per plant of *GSm* decreased most prominently, accounting for only 18.11% of WT. The second largest decrease occurred in main spike grain weight of *GSm*, which accounted for 25.94% of WT, and its thousand-grain weight accounted for 48.97% of WT. The results suggest that yield was seriously influenced by premature senescence in the *GSm* mutant (Table 1).

### 3.2. Differences in Physiological Traits between the Premature Senescence Mutant (GSm) and Its Wild-Type

**Figure 2:**
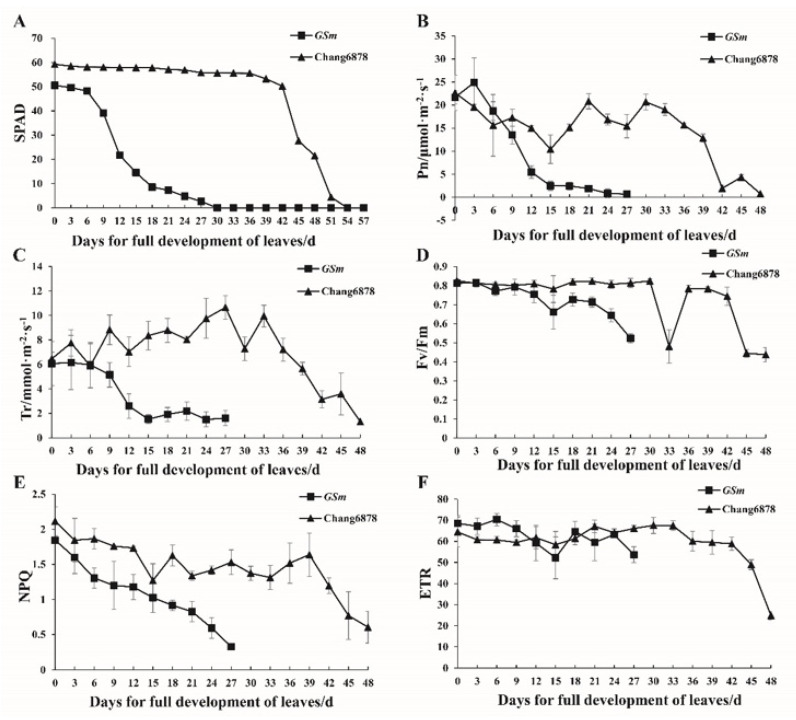
Photosynthetic fluorescence indices of flag leaf samples from the wheat premature senescence mutant (*GSm*) and its wild-type (WT). (**A**) Chlorophyll content; (**B**) Net photosynthetic rate, Pn; (**C**) Transpiration rate, Tr; (**D**) efficiency of primary conversion of light energy of photosystem II, Fv/Fm; (**E**) Non-photochemical quenching coefficient, NPQ; (**F**) Photosynthetic electron transport rate, ETR. Vertical bars represent standard errors with three independent biological replicates.

We analyzed chlorophyll content and photosynthetic fluorescence characteristics in flag leaves of WT and the *GSm* mutant from leaf full expansion to senescence and death. All SPAD values of WT were higher than those of *GSm* (Figure 2A). The analysis of senescence characteristic parameters indicated that senescence was initiated in fully unfolded flag leaves of *GSm* at 2.84 d, which was much earlier than 39.13 d in WT. Similarly, leaf senescence ended in *GSm* at 24.13 d, while this occurred in WT at 54.67 d (Table 2). Among the photosynthetic fluorescence indices of WT, the net photosynthetic rate (Pn), transpiration rate (Tr), efficiency of primary conversion of light energy of photosystem II (Fv/Fm), and non-photochemical quenching coefficient (NPQ) were all remarkably higher than those of *GSm* throughout the whole measurement period, except for the apparent photosynthetic electron transport rate (ETR; Figure 2B–F).

**Figure 3:**
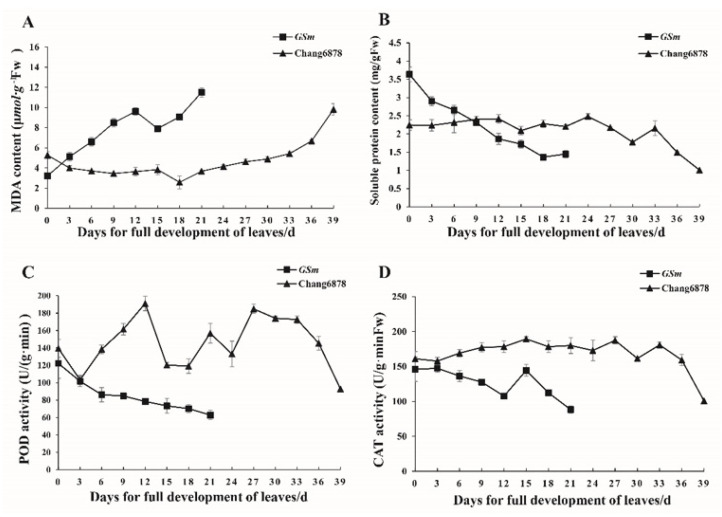
Senescence related physiological traits of *GSm* mutant and wild type. (**A**) Malondialdehyde content (**B**) soluble protein content (**C**) peroxidase activity (**D**) catalase activity. The vertical bar represents the standard error with three independent biological repetitions.

We also measured malondialdehyde (MDA) content, soluble protein content, peroxidase (POD) activity, and catalase (CAT) activity in flag leaves of WT and the *GSm* mutant during the senescence stage. MDA content in the flag leaves showed an overall upward trend in the wake of leaf senescence, and the increase was faster in *GSm* than in WT (Figure 3A). Conversely, soluble protein content displayed an overall downward trend, with considerably higher values for *GSm* compared with WT in the early stage of leaf full expansion; however, in the late stage, the rate of decrease was much faster in *GSm* than that in WT (Figure 3B). POD and CAT activity showed similar trends, both of which decreased faster in *GSm* compared with that in WT; yet, there was little change in CAT activity of WT, which decreased only in the late stage (Figure 3C,D).

### 3.3. RNA Sequencing Analysis and Identificaiton of Differentially Expressed Genes

A total of 12 sample libraries were constructed and sequenced, yielding 166.88 Gb of clean data (>11.79 Gb per sample). The GC content of raw reads in different libraries ranged between 50.79–54.88%, and the percentage of Q30 bases was >94.53% (Supplementary Table S1). The results indicate a high sequencing quality, so that the obtained data can be used for subsequent analysis of gene expression profiles and metabolic pathways.

We identified differentially expressed genes (DEGs) in *GSm* and WT during two different stages (Table 3). The flag leaves of *GSm* presented no senescence phenotype at 2 d after full expansion (S1 stage), but there were 7030 DEGs (5010 upregulated and 2020 downregulated) identified in WS1_vs_MS1. Then, a distinctive senescence phenotype was observed in the flag leaves of *GSm* at 9 d after full expansion (S2 stage), with 18,246 DEGs (10,801 upregulated and 7445 downregulated) identified in WS2_vs_MS2; meanwhile, there were 15,180 DEGs in MS1_vs_MS2 and 11,744 DEGs in WS1_vs_WS2.

**Figure 4:**
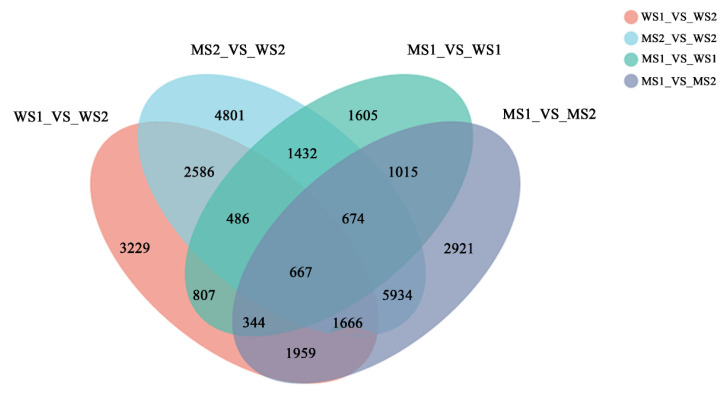
Venn diagram of DEG numbers in four samples.

Furthermore, the Venn diagram shows that based on the group comparisons of MS1_VS_WS1 and MS2_VS_WS2, there were 3771 and 14,987 unique DEGs in the S1 and S2 stages, respectively, with 3259 DEGs overlapped between the two libraries (Figure 4). This result suggests that distinctive response mechanisms were adopted in flag leaves of the mutant and WT during the two stages. Then, we randomly selected six genes from the DEGs and quantified their expression in different stages using quantitative reverse-transcription PCR (qRT-PCR). The qRT-PCR data showed that the six genes indeed differentially expressed, which was in agreement with the results of transcriptomic analysis (Supplementary Figure S1). The PCA of the different samples shows that there are differences between groups and little difference within groups (Supplementary Figure S2). Thus, the genome-wide transcriptomic analysis of wheat flag leaves was reliable.

### 3.4. GO Enrichment and KEGG Pathway Analysis of Differentially Expressed Genes

The Gene Ontology (GO) enrichment analysis of DEGs was carried out using the GO database (Table 4). For MS2_VS_WS2 and MS1_VS_MS2, the DEGs were enriched in GO terms of biological process, cellular component, and molecular function categories, mainly involving chlorophyll degradation (magnesium chelatase activity, xanthophyll biosynthetic process), photosynthesis (chloroplast thylakoid membrane protein complex, photosystem II oxygen evolving complex, assembly photosystem I reaction center), and respiration (fructose metabolic process, fructosyltransferase activity, fructose 1,6-bisphosphate 1-phosphatase activity, malate transmembrane transporter activity). For WS1_VS_WS2 and MS1_VS_WS1, the significantly enriched GO terms of DEGs were related to sugar metabolism (maltose metabolic process, fructosyltransferase activity, oligosaccharyltransferase complex) and amino acid metabolism (aromatic amino acid transmembrane transporter activity, S-adenosylhomocysteine metabolic process, S-adenosylhomocysteine metabolic process, asparaginase activity).

To identify the major pathways contributing to premature senescence, we analyzed the top five metabolic pathways obtained by different group comparisons using gene annotations in the Kyoto Encyclopedia of Genes and Genomes (KEGG) database (Table 5). The major enriched pathways of DEGs in the two groups of MS2_VS_WS2 and MS1_VS_MS2 were roughly the same and primarily related to chlorophyll degradation (porphyrin and chlorophyll metabolism, carotenoid biosynthesis), photosynthesis (photosynthesis-antenna proteins, carbon fixation in photosynthetic organisms), and respiration (glyoxylate and dicarboxylate metabolism, starch and sucrose metabolism). The DEGs in MS1_VS_WS1 were mainly enriched in the pathways related to protein processing and biosynthesis of different sugars (protein processing in endoplasmic reticulum, endocytosis, amino sugar and nucleotide sugar metabolism, N-glycan biosynthesis, various types of N-glycan biosynthesis). The DEGs in WS1_VS_WS2 were significantly enriched in the following pathways, glycerophospholipid metabolism; MAPK signaling pathway—plant; and glycine, serine and threonine metabolism.

### 3.5. Differentially Expressed Genes Involved in Chlorophyll and Carotenoid Biosynthesis and Photosynthesis

**Figure 5:**
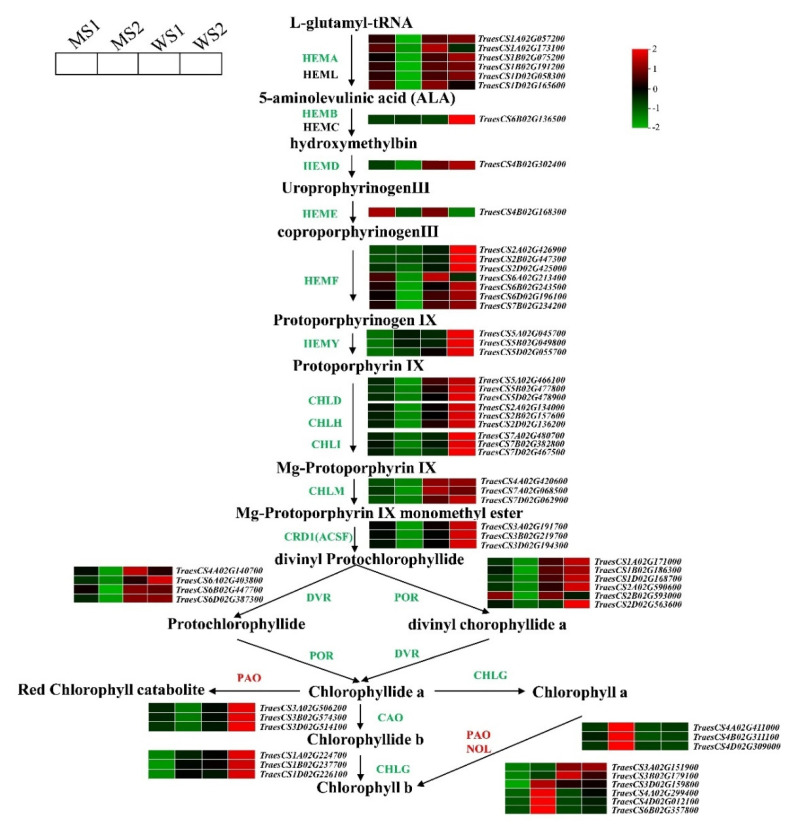
Chlorophyll metabolic pathway. Genes are shown in different colors. Relative gene expression levels are indicated by a color gradient from low (green) to high (red). Squares are arranged from left to right: MS1 (early senescence stage of mutant), MS2 (late senescence stage of mutant), WS1 (early senescence stage of wild-type), and WS2 (late senescence stage of wild-type). Numbers in the scale bar represent normalized transcripts per kilobase million (TPM) values of genes.

**Figure 6:**
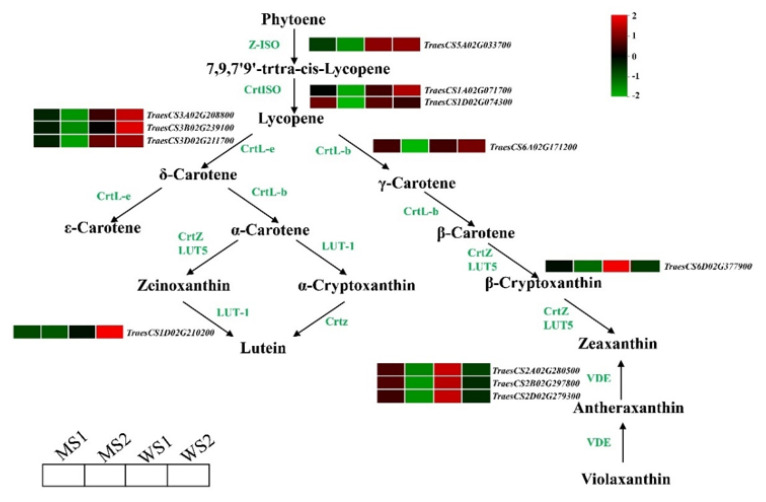
Carotenoid synthesis pathway.Genes are shown in different colors. Relative gene expression levels are indicated by a color gradient from low (green) to high (red). Squares are arranged from left to right: MS1 (early senescence stage of mutant), MS2 (late senescence stage of mutant), WS1 (early senescence stage of wild-type), and WS2 (late senescence stage of wild-type). Numbers in the scale bar represent normalized transcripts per kilobase million (TPM) values of genes.

To identify key genes associated with color formation of yellow leaves in the mutant and WT, we particularly compared their DEGs involved in chlorophyll metabolism and carotenoid biosynthesis pathways. We found that expression levels of 50 genes in chlorophyll biosynthesis pathway were downregulated in mutant leaves of the senescence stage (MS2; q < 0.05, fold-change > 2). However, there was a remarkable upregulation of DEGs that encode chlorophyll degradation-related enzymes, including three pheophytin oxygenase (*PAO*) genes and four NYC1-like (*NOL*) genes (Figure 5). An overall expression analysis revealed that compared with WT, most genes related to chlorophyll biosynthesis were expressed at lower levels in the mutant; however, there were no distinctive differences in expression levels of genes related to chlorophyll degradation in the MS1 stage compared with those of WS1 and WS2 (Supplementary Table S2). We also compared the DEGs involved in carotenoid biosynthesis and found a total of 12 genes in the entire metabolic pathway for lutein and zeaxanthin biosynthesis, whose expression levels were downregulated in the MS2 stage (Figure 6). An overall expression analysis indicated that compared with WT, expression levels of most genes related to carotenoid biosynthesis were also lower in the mutant in the two respective stages (Supplementary Table S3). With regard to photosynthetic antenna protein, the analysis results indicated that 77 genes were substantially downregulated in the MS2 stage, most of which showed lower expression levels in the mutant than in WT (Supplementary Table S4).

### 3.6. Differentially Expressed Genes Related to Antioxidative Metabolism and Protein Processing, Transportation

Antioxidative metabolism and cyanide-resistant respiration are critical to eliminate ROS accumulation and alleviate cellular damage during regulation of leaf senescence. The antioxidation-related DEGs identified in this study were mainly related to SOD, CAT, POD, and glutathione S-transferases (GSTs). Among them, two SOD-related genes, *TraesCS2A02G121200* and *TraesCS2D02G123300*, showed the highest expression levels compared with other related genes, and their expression levels were distinctively higher in the mutant than in WT. With regard to CAT, most genes in the mutant were expressed at higher levels in the senescence stage (MS2) than in the non-senescence stages (MS1, WS1, and WS2). As for GSTs and POD, some genes exhibited the same expression patterns, with considerably higher expression levels in the mutant compared with those of WT. Another portion of the genes related to GSTs and POD were expressed at higher level in the senescence stage than in the non-senescence stages, similar to the results for CAT-related genes. Only a few genes were highly expressed in WT (Supplementary Table S5). Analyzed the DEGs of the 5 main enriched pathways in the MS1_VS_WS1 stage and found that there were 93 DEGs in total, of which 81 were down-regulated and only 12 were up-regulated, indicating that protein processing and transport were enhanced in the mutant at this stage. This may result in higher protein levels in the mutant than in the wild type.

### 3.7. Differentially Expressed Genes Associated with Hormone Signaling

**Figure 7:**
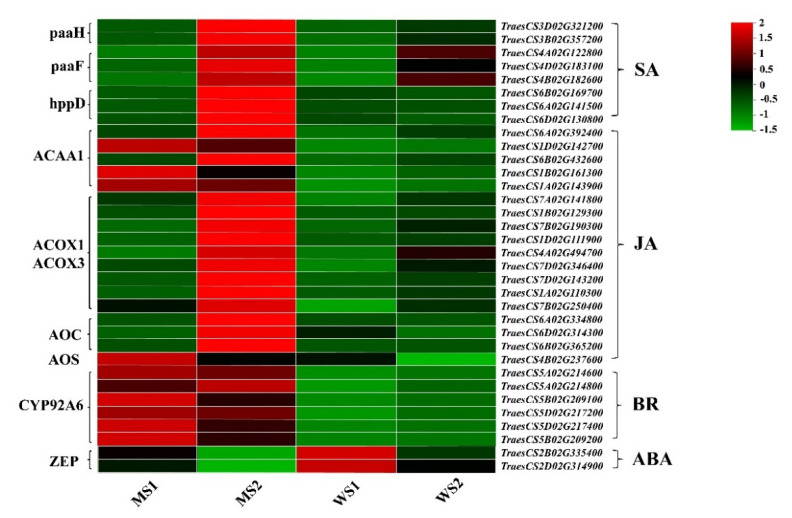
Expression profile clustering of genes involved in different hormone pathways. The expression ratio is standardized based on normalized transcripts per kilobase million (TPM) values, where each vertical column represents the mean of three samples (MS1, MS2, WS1 and WS2), and each horizontal row represents a sample single gene.

We analyzed hormone biosynthesis-related DEGs between the mutant and WT, finding that these genes mainly participated in the biosynthesis of salicylic acid (SA), JA, and brassinolides (BR). Most of the DEGs displayed different increases in their transcript abundances in *GSm* during leaf senescence, especially in the MS2 stage. Expression levels of SA- and JA-related biosynthetic genes were all higher in the MS2 stage than in the MS1, WS1, and WS2 stages. Additionally, 10 ACAA1- and BR-related biosynthetic genes (e.g., *TraesCS5A02G214600*, *TraesCS5A02G214800*, *TraesCS5B02G209100*, *TraesCS5D02G217200*, *TraesCS5D02G217400*, and *TraesCS5B02G209200*) were expressed at higher levels in the mutant compared with those in WT. In the case of ABA, we found that the DEGs encoding zeaxanthin epoxidase were markedly downregulated in the MS2 stage (Figure 7). However, no major changes occurred in the transcript abundance of CK- and GA-related DEGs between the two materials (Supplementary Table S6).

### 3.8. Differentially Expressed Genes Participating in Autophagy and Hydrolysis of Senescent Leaves

**Figure 8:**
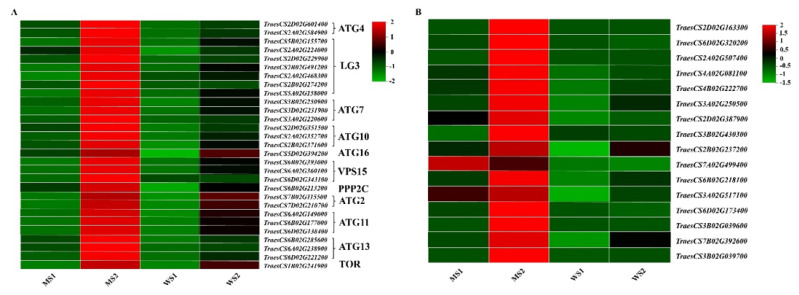
Expression profile clustering of autophagy related genes. The expression ratio is standardized based on normalized transcripts per kilobase million (TPM) values, where each vertical column represents the mean of three samples (MS1, MS2, WS1 and WS2), and each horizontal row represents a sample single gene.

Autophagy plays an essential role in maintaining normal life activities of cells. A total of 29 DEGs encoding autophagy-related proteins (e.g., *ATG4*, *LC3*, *ATG7*, *ATG10*, and *ATG16*) were detected. Their transcription in the leaves of *GSm* was considerably promoted in the MS2 stage (Figure 8A). In addition, there were 16 DEGs mainly participating in hydrolysis and autophagy; these genes showed the same expression patterns, with expression levels being elevated in the *GSm* mutant during leaf senescence (Figure 8B). These results show that autophagy- and hydrolysis-related genes might play an indispensable regulatory role in macromolecular hydrolysis and cell apoptosis in the *GSm* mutant during leaf senescence.

### 3.9. Transcription Factor Family Members in Differentially Expressed Genes

**Figure 9:**
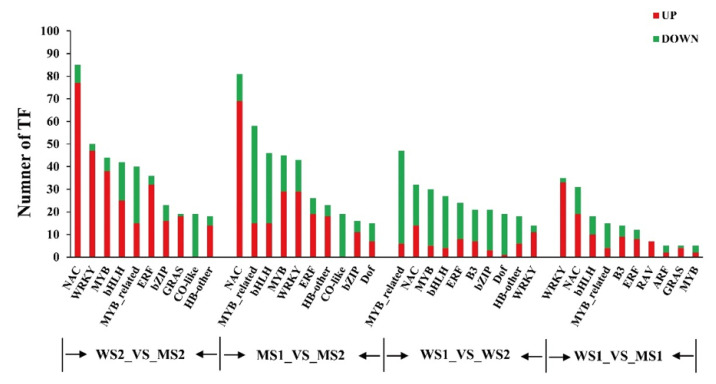
Statistics of the number of different transcription factors involved in the activity of sequence specific DNA binding transcription factors under the comparison of four groups.

**Figure 10:**
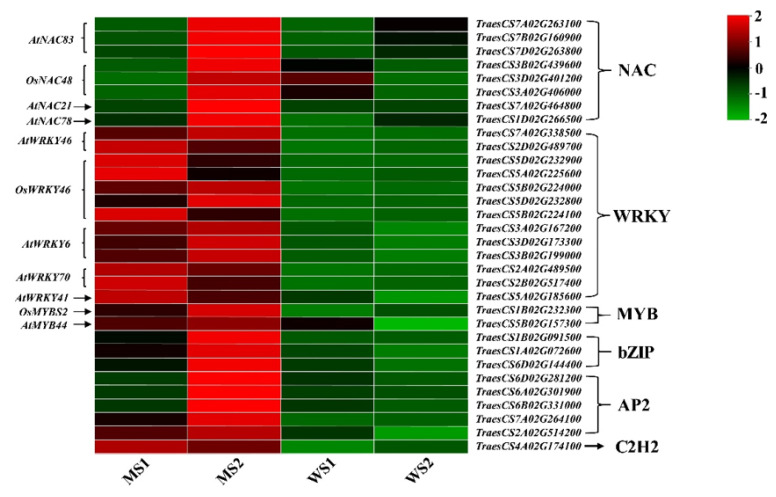
Expression profile clustering of transcription factor related genes. The expression ratio is standardized based on normalized transcripts per kilobase million (TPM) values, where each vertical column represents the mean of three samples (MS1, MS2, WS1 and WS2), and each horizontal row represents a sample single gene.

We detected 358, 520, 181, and 494 transcription factors that were differentially expressed in the group comparisons of WS1_VS_WS2, MS2_VS_WS2, MS1_VS_WS1, and MS1_VS_MS2, respectively. The number of differentially expressed transcription factors was relatively high in the group comparisons containing MS2 stage, indicating that more transcription factors took part in the regulation of leaf senescence (Table 6). Then, we identified the top 10 differentially expressed transcription factors in the four group comparisons. The numbers of upregulated genes were more than those of downregulated genes for most transcription factors in the senescence stage (MS1_VS_MS2 and MS2_VS_WS2). Of these, the numbers of upregulated NAC genes were the highest, 77 and 69, which were much higher than the numbers of upregulated genes before senescence, 14 and 19 (WS1_VS_WS2 and MS1_VS_WS1). The second largest class was *WRKY* and *MYB*, followed by *ERF*, all of which had a considerably higher number of upregulated genes after senescence than before senescence. The Co-like family genes were all downregulated in the senescence stage (Figure 9). We identified the transcription factors with relatively high transcript abundance, most of which belonged to the NAC, WRKY, MYB, and AP2 families. These genes showed much higher expression levels in senescent leaves of the mutant (MS2) compared with those of non-senescent mutant (MS1) and WT (WS1 and WS2) leaves. Moreover, *WRKY* and *MYB* expression levels were both higher in the mutant than in WT. Furthermore, despite the total number of *C2H2* genes in the four groups was not high, their transcript abundances in the mutant were higher compared with those of WT. Thus, these transcription factors played a significant role in premature leaf senescence of the *GSm* mutant (Figure 10).

## 4. Discussion

### 4.1. Agronomic, Yield, and Physiological Traits Are Distinctively Different between the Common Wheat Premature Senescence Mutant and Its Wild-Type

Using mutagenesis techniques to obtain stable mutant materials is essential for wheat breeders to explore the physiological mechanisms of wheat growth and development, and to cultivate new cultivars with high and stable yield and strong stress resistance. Here, we used EMS to mutate the common wheat cultivar Chang 6878 and then acquired the premature senescence mutant *GSm* with a stable phenotype after multiple generations of selection. Compared with WT, the chlorophyll content in flag leaves of *GSm* decreased substantially throughout the whole growth period, whereas the photosynthetic fluorescence-related indices (Pn, Tr, Fv/Fm, NPQ, and ETR) all decreased distinctively after full expansion of flag leaves. In contrast, MDA content increased faster in *GSm* than in WT. Soluble protein content in *GSm* was also distinctively higher than that of WT in the early stage of leaf full expansion, but a remarkable decrease occurred in the late stage. Both POD and CAT activity showed a faster decrease in the mutant than in WT. Furthermore, agronomic traits, such as effective tillering, plant height, Panicle length, number of grains per panicle per spike, number of fruiting spikelets, Main spike grain weight, yield per plant, and thousand-grain weight, all prominently decreased in the mutant compared with WT. The results corroborate previous studies reported by Li et al. [38] and Zhang et al. [40], indicating that premature senescence indeed has a profound influence on common wheat yield.

### 4.2. Photosynthesis-Related Genes Are Differentially Expressed in the Leaves of Common Wheat Premature Senescence Mutant and Its Wild-Type

Premature senescence of leaves is closely linked to chlorophyll metabolism and carotenoid metabolism. In a wheat premature senescence mutant, *Ygm*, expression levels of eight genes encoding magnesium chelatase H subunit and protochlorophyllide oxidoreductase in the chlorophyll metabolic pathway are downregulated, whereas two genes encoding β-carotene hydroxylase in the carotenoid biosynthetic pathway are upregulated [39]. In addition, a rice pale-green leaf mutant, *pgl10*, shows remarkably decreased content chlorophyll (chlorophyll a and b) and carotenoid, with expression of chlorophyll biosynthesis-related genes being lowered; bioinformatic analysis indicates that *PGL10* encodes protochlorophyllide oxidoreductase B [46]. Moreover, virus-induced gene silencing of *CHLI* and *CHLD* genes produces a yellow leaf phenotype in pea, which exhibits diminished chlorophyll accumulation and considerably decreased photosynthetic protein levels [12]. The rice *OsCHLH* gene encodes the largest subunit of magnesium chelatase, a key enzyme in the chlorophyll branch of the tetrapyrrole biosynthesis pathway. Abnormal chloroplast development and low chlorophyll content have been found in a rice T-DNA insertion mutant, *OsCHLH* [47].

Levulinate, an inhibitor of 5-aminolevulinate dehydratase, can be used to inhibit the biosynthesis of pyrrole-derived tetrapyrrole chlorophyll. Expression levels of nuclear genes involved in carotenoid biosynthesis (i.e., geranylgeranyl diphosphate synthase, phytoene synthase, and phytoene desaturase) are downregulated in levulinate-treated seedlings. Likewise, transcript abundances of nuclear genes encoding chloroplast proteins (i.e., *Lhcb1*, *PsbO*, and *RcbS*) are severely decreased in levulinate-treated samples [48]. Map-based cloning of a rice premature senescence mutant, eas1, reveals that the nuclear gene *EAS1* encodes PaO [49]. *TaPAO* expression level is upregulated and Fv/Fm is lowered distinctively in the wheat mutant *m68* [40]. In the present study, we compared the DEGs involved in chlorophyll metabolism and carotenoid biosynthesis pathways between the premature senescence mutant *GSm* and WT of common wheat. We found that compared with WT, expression levels of genes related to the biosynthesis of chlorophyll, lutein, zeaxanthin, and photosynthesis-antenna proteins in the mutant were all substantially downregulated in the S1 and S2 stages; conversely, the DEGs encoding chlorophyll degradation-related enzymes (PAO and NOL) were upregulated remarkably in the MS2 stage. The results suggest blockade of chlorophyll and carotenoid biosynthesis in the *GSm* mutant, which caused a decrease in its photosynthetic capacity and hence accelerated chlorophyll degradation in the late stage of leaf senescence. Further, we compared the chlorophyll synthesis-related genes in the leaf senescence process of common wheat (Supplementary Figure S3) that was analyzed during the natural senescence process of common wheat variety Jinmai39 in our lab (unpublished data) and premature senescence mutant *Ygm* [39] with the *GSm* mutants. It was found that except for HEML and HEMC, rest of the chlorophyll synthesis-related genes were significantly down-regulated in *GSm* mutant, while only a few genes in common wheat and *Ygm* were down-regulated. These results indicated that although the plants are all senescent, the chlorophyll synthesis-related genes regulated by the senescence process were significantly different in *GSm* mutant. Therefore, *GSm* mutant specifically inhibited the chlorophyll synthesis and accelerated chlorophyll degradation that influence premature senescence in wheat.

### 4.3. Antioxidant System-Related Genes Are Differentially Expressed between the Common Wheat Premature Senescence Mutant and Its Wild-Type 

ROS are generated in cells as an inevitable result of aerobic metabolism [50]. The generation and elimination of ROS in plant cells are in equilibrium under normal conditions owing to activation of antioxidant enzymes. However, ROS may accumulate in degenerated tissues, with antioxidant enzyme activity gradually lost during senescence [51,52]. Two rice premature senescence mutants, *PLS2* and *psls1*, show considerably diminished CAT activity, enhanced H_2_O_2_ accumulation, and increased dead cells [53,54]. A premature senescence and death 128 mutant (*psd128*) isolated from the rice *IR64* mutant library exhibits markedly decreased soluble protein content and increased MDA content [33,46]. Through comparing near-isogenic lines of wheat with premature and normal senescence, a study has found that the enzyme activity of the antioxidant system, such as superoxide dismutase (SOD), CAT, ascorbate peroxidase, and glutathione reductase, is distinctively inhibited, and the redox system is destructed, which may lead to premature leaf senescence [55].

Similar to the previous studies, we found that compared with WT, MDA content increased faster and soluble protein content decreased faster in the flag leaves of the common wheat premature senescence mutant *GSm*. Additionally, POD and CAT activity displayed similar trends, both decreasing faster in the mutant than in WT. Further, transcriptomic analysis revealed that antioxidation-related DEGs were primarily associated with biosynthesis of SOD, CAT, POD, and GSTs. In particular, two related genes encoding SOD1 (*TraesCS2A02G121200* and *TraesCS2D02G123300*) and other related genes were expressed at considerably higher levels in the mutant compared with WT. Expression levels of most genes related to CAT in the mutant were higher in the senescence stage (MS2) than in the non-senescence stages (MS1, WS1, and WS2). This also suggests that premature senescence in the mutant results in substantial H_2_O_2_ production, thereby triggering expression of CAT-related genes. As for GSTs and POD, the same expression patterns were observed in some genes, whose expression levels were distinctively higher in the mutant compared with WT. Accordingly, the *GSm* mutant may generate more ROS in the early stage of leaf senescence, so that multiple antioxidant enzymes work together to minimize ROS-induced oxidative damage.

### 4.4. Plant Hormones and Autophagy Play an Essential Regulatory Role in Leaf Senescence

Many studies indicate that Salicylic acid, Jasmonic acid, Brassinolide, abscisic acid, ethylene, and Strigolactones facilitate the senescence process, whereas IAA, GA, and cytokinins (CKs) play a role in delaying senescence in plants. Approximately 60% of SA biosynthesis and signaling genes are upregulated in senescent leaves of *A. thaliana* [56]. *Teosinte Branched/Cycloidea/PCF4* (*TCP*) transcription factors play a bifunctional role in integrating JA signal into plant senescence process. Activation of *tcp* and *tcp20* inhibits biosynthesis of lipoxygenase 2, which reduces JA accumulation and ultimately inhibits plant senescence [57]. In contrast, *tcp4* activates lipoxygenase 2 biosynthesis, leading to an elevation in JA levels, which in turn accelerates plant senescence [58]. Additionally, a mutant unsusceptible to exogenous, treatment shows decreased senescence-associated gene transcript levels, so that its senescence is delayed [59]. Conversely, *BRI1-EMS-suppressor 1* (*BES1*) is shown to accelerate plant senescence because of an upregulation in the BR response pathway [60]. Furthermore, the underlying mechanisms by which ABA induces and accelerates plant senescence have been reported in many studies. Exogenous application of ABA can increase the accumulation of H_2_O_2_ in plant cells [61]. ABA can also induce expression of antioxidant enzymes such as SOD and CAT to participate in plant senescence [62]. Meanwhile, *ABA-inducible receptor kinase* (*RPK1*) gene shows upregulated expression in *A. thaliana* during the senescence process [63].

In the present study, we analyzed hormone biosynthesis-related DEGs between the common wheat premature senescence mutant *GSm* and its WT. Most of the DEGs were found to show different increases in their transcript abundances in the mutant during leaf senescence. This result indicates an enhancement of hormone signaling pathway in senescent leaves of *GSm*, especially during the MS2 stage. Additionally, the biosynthetic genes related to SA and JA all had higher expression levels in the MS2 stage than in the MS1, WS1, and WS2 stages. However, there were no major changes in transcript abundances of CK- and GA-related DEGs between the two materials, suggesting that the signaling pathways corresponding to CK and GA may play a minor regulatory role in leaf senescence. The genes related to ABA biosynthesis were downregulated in the mutant, possibly because ABA mainly plays its role in the late senescence stage. All the six biosynthetic genes related to BR showed higher expression levels in the mutant compared with those of WT. SA was reported to be significantly increased in *Arabidopsis*, mainly during senescence with higher chlorophyll degradation, suggesting that SA is involved in regulating senescence [64]. Studies have shown that JA production is downregulated early in aging, and later integrates signals from external and internal factors, ultimately leading to the onset of aging [25]. It has been reported BR can accelerate the degradation of chlorophyll [65], but no studies have shown that it plays a role in the early stage of senescence. Therefore, our data suggested that genes from SA, JA and BR signaling pathways might be affected by *GSm* mutant which accelerates premature senescence in wheat.

Autophagy plays a vital role in cell development and differentiation, maintenance of normal cell life activities, resistance to senescence, and defense against abiotic stress and pathogen invasion [31,32]. Previously, four DEGs encoding autophagy-related proteins (i.e., 3, 8A, 8B, and 8C) were detected in a rice premature senescence mutant, *ospls1*. Their transcription was considerably promoted in the leaves of the *ospls1* mutant during the grain filling stage [33]. Here, we identified a total of 29 DEGs associated with autophagy in senescent leaves, including genes encoding autophagy-related proteins ATG4, LC3, ATG7, ATG10, and ATG16. Expression levels of these DEGs in the *GSm* mutant were elevated during leaf senescence. Hydrolysis-related genes had the same expression patterns as autophagy-related genes; their expression levels were markedly upregulated in senescent leaves of the mutant. These results suggest that autophagy-related proteins and proteasomes may play an integral role in regulating macromolecular hydrolysis and cell apoptosis in the *GSm* mutant during leaf senescence.

### 4.5. Transcription Factors Play a Vital Role in Senescence Regulation in Common Wheat

Transcription factors are proteins that activate or repress gene expression through binding to cis-regulatory elements in gene promoters. Transcription factors play a crucial role in plant leaf senescence [38,66]. Senescence-related gene regulatory networks in *A. thaliana*, rice, and wheat reveal the vital role of transcription factor families NAC and WRKY [67,68,69,70]. The senescence regulatory network in wheat contains differentially expressed transcription factors and is mainly enriched for *NAC* (61), *MYB*-associated (43), *WRKY* (27), and *AP2/EREBP* (16) genes. We found that the number of differentially expressed transcription factors also increased sequentially with the progression of leaf senescence, which indicates that more transcription factors participate in senescence regulation in common wheat. The upregulated genes in the senescence stage were mainly identified as *NAC*, *WRKY*, and *MYB*.

The rice *OsNAP* gene can directly induce expression of genes involved in chlorophyll degradation, and *OsNAP*-overexpressing plants present a premature senescence phenotype [71]. In addition, *NAC83* gene expression can be induced by ABA, drought, low temperature, and high salt stress, while expression differences occur in different citrus species. Grapefruit (*Citrus maxima* [Burm] Merr.) *CmNAC83*, trifoliate orange (*Poncirus trifoliata* [L.] Raf) PtNAC83, and lemon (*Citrus limon* [L.] Burm. f.) *ClNAC83* are members of the *NAC* gene family, all of which perform essential functions in the abiotic stress response of citrus [72]. *TaNACA* expression in wheat flag leaves increases with the procession of leaf senescence, and heterologous overexpression of *TaNACA* into *A. thaliana* causes evident premature leaf senescence. *ZmNAC48* participates in plant drought stress response in corn. Moreover, *ZmNAC48*-overexpressing *A. thaliana* has improved drought tolerance, reduced water loss rate, enhanced stomatal closure, and increased plant survival rate [73]. Xu, Huang, and Ning et al. showed that wheat *TaNAC29*, *TaNAC2D*, and *TaNAC4* genes all positively regulate plant leaf senescence [74,75,76]. Furthermore, high expression of *GhNAC78* occurs in cotton (*Gossypium hirsutum*) leaves during the senescence process; so, this gene may be involved in the regulation of leaf senescence [77]. In the present study, we observed high expression of *NAC83*, *NAC48*, and *NAC78* genes in MS2, suggesting that the *GSm* mutant may be under positive regulation by these genes in the S2 stage, leading to accelerated leaf senescence.

*BcWRKY46* from *Brassica campestris* ssp. chinensis Makino reportedly performs crucial functions in tobacco in response to ABA and abiotic stress [78]. *WRKY46* expression is rapidly induced by drought, salt, and oxidative stress in *A. thaliana*, and it modulates a group of genes involved in cellular osmoprotection and redox homeostasis under dehydration stress [79]. *A. thaliana WRKY46* expression is also induced specifically by SA [80]. Additionally, *MtWRKY76* expression is induced rapidly by abiotic stress in alfalfa (*Medicago truncatula* L.); overexpression of *MtWRKY76* distinctively enhances salt and drought tolerance in transgenic alfalfa plants and triggers abiotic stress-inducible genes [81]. The sunflower (*Helianthus annuus* L.) transcription factor *HaWRKY76* confers tolerance to dehydration and waterlogging in transgenic *A. thaliana* plants without causing yield loss [82]. *WRKY6* regulates many senescence-related genes, including senescence-induced receptor-like gene kinase and pathogenesis-related genes [83]. *OsWRKY6* is a positive transcriptional regulator of plant defense in rice [84] and *A. thaliana* [85]. *AtWRKY70* from *A. thaliana* performs its functions downstream of defense-related ROS intermediates and SA [86,87]. *A. thaliana* transcription factors WRKY46, WRKY54, and WRKY70 participate in brassinosteroid-regulated plant growth and drought responses [88]. Expression of *HbWRKY41* in rubber (*Hevea brasiliensis* Muell. Arg.) is upregulated by cold, drought, and salt stress, and its expression increases continuously under cold or salt stress; thus, *HbWRKY41* may be a major regulator of leaf senescence and abiotic stress [89]. Furthermore, the MYB transcription factor *OsMYBS1* reportedly takes part in glucose and hormonal regulation [90,91]. We found that *WRKY46*, *WRKY76*, *WRKY6*, *WRKY70*, and *WRKY41*, together with *MYBS2* and *MYB44*, all showed high expression in the S1 and S2 stages; these genes were all induced to express in the mutant earlier than *NAC* genes. The *WRKY* and *MYB* gene expression is likely to be triggered by higher levels of ROS and plant hormones (e.g., BR, SA, and JA) in the *GSm* mutant, thereby accelerating leaf senescence. Overall, transcription factors play a crucial role in the premature senescence of mutants, especially WRKY and MYB. However, these transcription factors are specific to the *GSm* mutant or involve in the natural leaf senescence process that needs to be further investigated.

## 5. Conclusions

This study found that compared with the WT of common wheat, leaf senescence was accelerated and grain yield was decreased substantially in a premature senescence mutant, *GSm*. *GSm* and WT showed distinctive differences in their transcriptome profiles. Different genes related to premature leaf senescence were identified, showing blockade of chlorophyll and carotenoid biosynthesis, accelerated chlorophyll degradation, and diminished photosynthetic capacity in mutant leaves. In addition, the antioxidant system played a role to minimize oxidative damage in the mutant. There was an upregulation of SA, JA, and BR biosynthesis-related genes in the senescence stage. In particular, BR-related genes were most prominently upregulated in the mutant during the non-senescence stage, indicating that this hormone may increase chlorophyll breakdown and thereby accelerate leaf senescence. *NAC83*, *NAC48*, and *NAC78* positively regulated leaf senescence. Compared with the *NAC* genes, *WRKY46*, *WRKY76*, *WRKY6*, *WRKY70*, and *WRKY41*, as well as *MYBS2* and *MYB44* were induced to express earlier in the mutant. The *WRKY* and *MYB* gene expression was possibly triggered by increased levels of ROS and plant hormones (e.g., BR, SA, and JA) in the mutant, which in turn accelerated leaf senescence. Taken together, the results shed light on the molecular mechanisms of premature leaf senescence in common wheat.

## Figures and Tables

**Table 1:** The main agronomic traits of the WT and mutant.

Agronomic Traits	Wild Type	Mutant
Effective tiller/plant	6.4 ± 1.96	2.60 ± 0.49 *
Plant height (cm)	62.12 ± 3.3	49.40 ± 2.87 *
NO. of upper sterile spikelets	0 ± 0	0.20 ± 0.4
NO. of lower sterile spikelets	0 ± 0	1.60 ± 0.49 *
Panicle length (cm)	14.56 ± 0.29	9.78 ± 0.72 *
NO.of grains per panicle	100.40 ± 9.02	33.80 ± 2.79 *
NO. of fruiting spikelets	23.40 ± 0.49	16.60 ± 0.80 *
Main spike grain weight (g)	2.93 ± 0.55	0.76 ± 0.23 *
Grain yield per plant (g)	13.75 ± 4.40	2.49 ± 0.73 *
1000-grainweight (g)	30.16 ± 0.53	14.77 ± 0.33 *
Flag leaf length (cm)	15.75 ± 1.89	16.06 ± 1.28
Flag leaf width (cm)	1.5 ± 0.16	1.32 ± 0.12 *
Second leaf length (cm)	20.66 ± 2.64	20.44 ± 1.30
Second leaf width (cm)	1.38 ± 0.11	1.22 ± 0.07 *

Dates are the mean ± standard deviation of three repetition. * is significant difference compare with wild type (*p* < 0.05).

**Table 2:** Aging characteristic parameters of wild type and mutant.

Varirty	Curve Parameters			Characteristic			
	k	a	b	MRS	TMRS/d	Ts/d	To/d
*GSm*	63.047	0.1047	−0.1404	3.26	16.07	2.84	24.13
Chang6878	57.781	1.82 × 10^−6^	−0.2618	5.56	50.48	39.13	54.67

**Table 3:** DEGs identified from five different comparisons. DEGs: differentially expressed genes.

DEG-Set	Total DEG	Up-Regulated	Down-Regulated
WS2_VS_MS2	18,246	10,801	7445
MS1_VS_MS2	15,180	8246	6934
WS1_VS_MS1	7030	5010	2020
WS1_VS_WS2	11,744	5145	6599

**Table 4:** Main enrichment pathways of DEG in 4 groups, refer to go database.

Sample Pair	Class	Annotation	GO ID	*p*-Value
WS1_VS_WS2	Biological process	maltose metabolic process	GO:0000023	1.19 × 10^−^^7^
		tertiary alcohol metabolic process	GO:1902644	3.1 × 10^−7^
	Cellular component	plastoglobule	GO:0010287	5.92 × 10^−7^
		cytoskeleton	GO:0005856	1.13 × 10^−6^
	Molecular function	aromatic amino acid transmembrane transporter activity	GO:0015173	1.19 × 10^−7^
		fructosyltransferase activity	GO:0050738	2.78 × 10^−7^
MS2_VS_WS2	Biological process	photosystem II oxygen evolving complex assembly	GO:0010270	4.55 × 10^−8^
		xanthophyll biosynthetic process	GO:0016123	1.5 × 10^−7^
	Cellular component	cell surface	GO:0009986	6.12 × 10^−8^
		chloroplast thylakoid membrane protein complex	GO:0098807	9.89 × 10^−8^
	Molecular function	magnesium chelatase activity	GO:0016851	4.55 × 10^−8^
		malate transmembrane transporter activity	GO:0015140	4.55 × 10^−8^
MS1_VS_WS1	Biological process	S-adenosylhomocysteine metabolic process	GO:0046498	4.58 × 10^−8^
		S-adenosylhomocysteine catabolic process	GO:0019510	4.58 × 10^−8^
	Cellular component	COPI-coated vesicle membrane	GO:0030663	7.61 × 10^−8^
		oligosaccharyltransferase complex	GO:0008250	1.63 × 10^−7^
	Molecular function	asparaginase activity	GO:0004067	7.61 × 10^−8^
		protein disulfide isomerase activity	GO:0003756	3.15 × 10^−7^
MS1_VS_MS2	Biological process	xanthophyll metabolic process	GO:0016122	1.45 × 10^−7^
		fructose metabolic process	GO:0006000	2.16 × 10^−7^
	Cellular component	chloroplast thylakoid membrane protein complex	GO:0098807	1.62 × 10^−7^
		photosystem I reaction center	GO:0009538	1.66 × 10^−7^
	Molecular function	fructosyltransferase activity	GO:0050738	7.88 × 10^−8^
		fructose 1,6-bisphosphate 1-phosphatase activity	GO:0042132	1.62 × 10^−7^

**Table 5:** Main enrichment pathways of DEG in 4 groups, refer to KEGG database.

Sample Pair	Pathway	Ko ID	*p*-Value
WS1_VS_WS2	Starch and sucrose metabolism	map00500	8.02 × 10^−11^
	Glycerophospholipid metabolism	map00564	5.49 × 10^−11^
	Carotenoid biosynthesis	map00906	1.9 × 10^−10^
	MAPK signaling pathway—plant	map04016	1.12 × 10^−8^
	Glycine, serine and threonine metabolism	map00260	6.23 × 10^−8^
MS2_VS_WS2	Photosynthesis—antenna proteins	map00196	9.69 × 10^−45^
	Porphyrin and chlorophyll metabolism	map00860	4.92 × 10^−24^
	Glyoxylate and dicarboxylate metabolism	map00630	2.83 × 10^−14^
	Carbon fixation in photosynthetic organisms	map00710	8.26 × 10^−14^
	Carotenoid biosynthesis	map00906	3.22 × 10^−11^
MS1_VS_WS1	Protein processing in endoplasmic reticulum	map04141	3.25 × 10^−25^
	Endocytosis	map04144	8.51 × 10^−14^
	Amino sugar and nucleotide sugar metabolism	map00520	2 × 10^−13^
	N-Glycan biosynthesis	map00510	3.3 × 10^−11^
	Various types of N-glycan biosynthesis	map00513	1.09 × 10^−10^
MS1_VS_MS2	Photosynthesis—antenna proteins	map00196	2.23 × 10^−44^
	Carbon fixation in photosynthetic organisms	map00710	1.9 × 10^−25^
	Starch and sucrose metabolism	map00500	3.75 × 10^−25^
	Glyoxylate and dicarboxylate metabolism	map00630	3.15 × 10^−20^
	Porphyrin and chlorophyll metabolism	map00860	5.48 × 10^−19^

**Table 6:** Transcription factor statistics of 4 groups.

Sample Pair	UP TF Number	DOWN TF Number	ALL TF Number
WS1_VS_WS2	96	262	358
MS2_VS_WS2	358	162	520
MS1_VS_WS1	111	70	181
MS1_VS_MS2	256	238	494

## Data Availability

The data presented in this study are openly available in NCBI (https://www.ncbi.nlm.nih.gov/bioproject/, reference number PRJNA823852, accessed on 19 April 2022).

## Supplementary Materials

The following supporting information can be downloaded at: https://www.mdpi.com/article/10.3390/biology11060904/s1, Figure S1: Some important differentially expressed genes verified using qRT-PCR. The error bars represent the SD of the means (n = 3); Figure S2: PCA of the different samples; Figure S3: chlorophyll metabolic pathway of common wheat (Green indicates down-regulated expression and red indicates up-regulated expression). RNA-seq was done in the flag leaf of Common wheat variety Jinmai 39 and leave samples were collected at 0 d, 26 d and 30 d after flowering; Table S1: Quality and Mapping reads of RNA-Seq; Table S2: List of Chlorophyll-related DEGs identified in WT and *GSm*; Table S3: List of carotenoid synthesis related DEGs identified in WT and *GSm*; Table S4: List of photosynthesis related DEGs identified in WT and *GSm*; Table S5: List of enzyme activity-related DEGs identified in WT and *GSm*; Table S6: List of cytokinin and gibberellin-related DEGs identified in WT and *GSm*; Table S7: Primers for quantitative real-time polymerase chain reaction.
