# Supplementary material for: Analysis of Physiological and Transcriptomic Differences between a Premature Senescence Mutant (GSm) and Its Wild-Type in Common Wheat (Triticum aestivum L.)
Source: Biology (Basel). 2022 Jun 12;11(6):904. doi: 10.3390/biology11060904 (PMC9219967; doi:10.3390/biology11060904)
Supplement: Supplementary file 1 [file biology-11-00904-s001.zip › biology-1722928-supplementary.pdf]

Supplementary Materials:

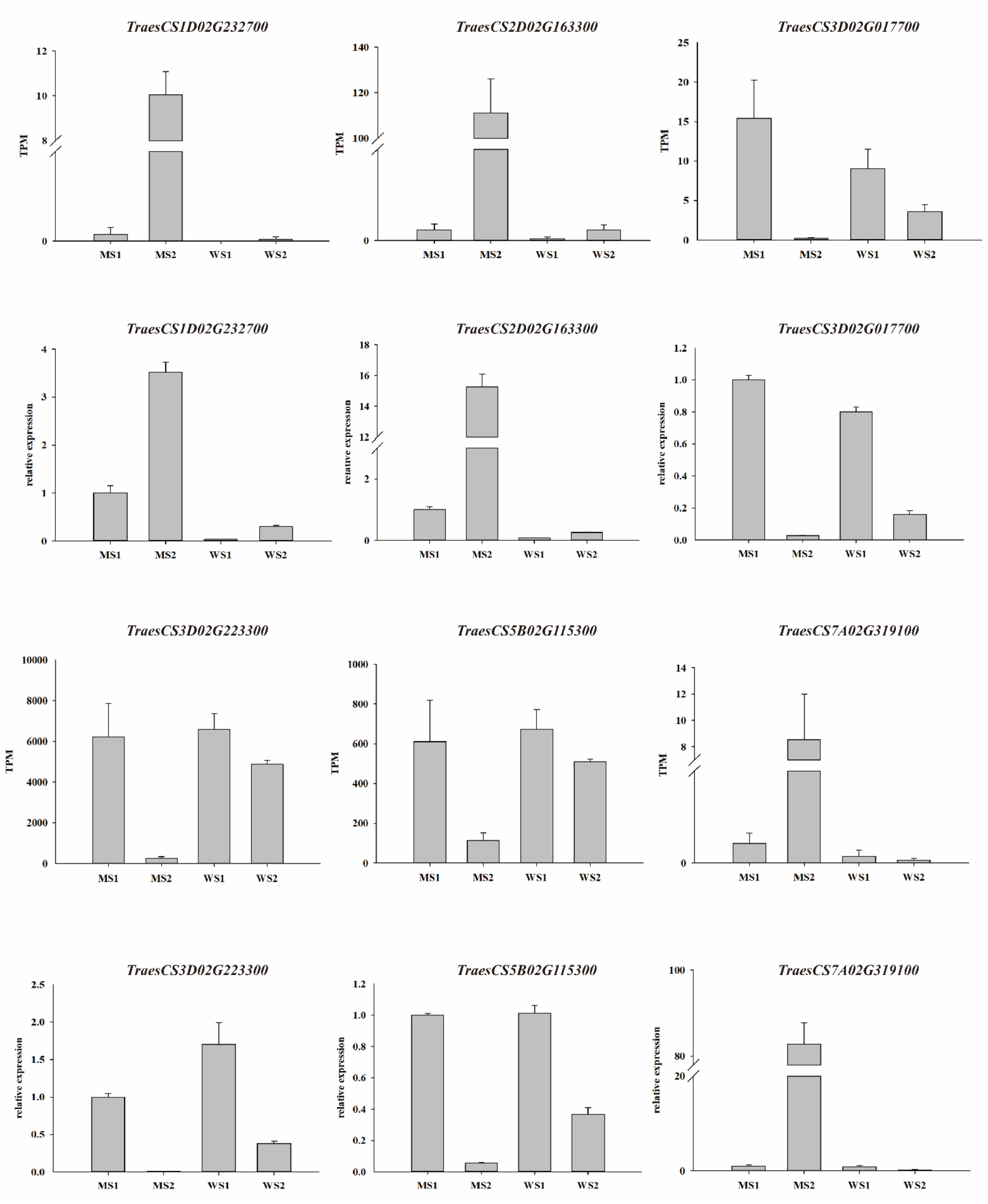

Figure S1. Some important differentially expressed genes verified using qRT-PCR. The error bars represent the SD of the means (n=3).

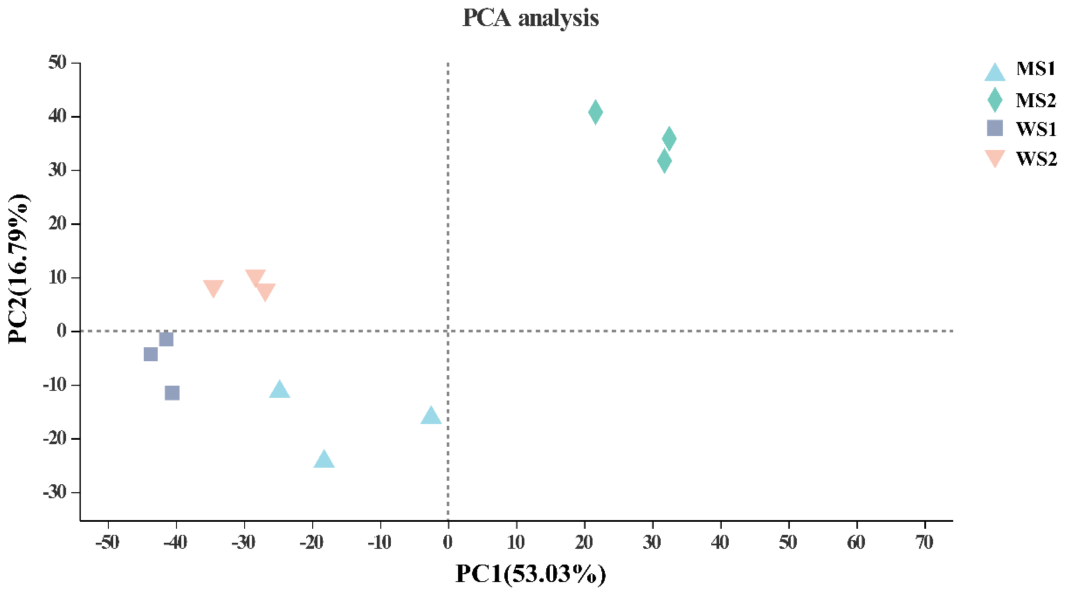

Figure S2. PCA of the different samples

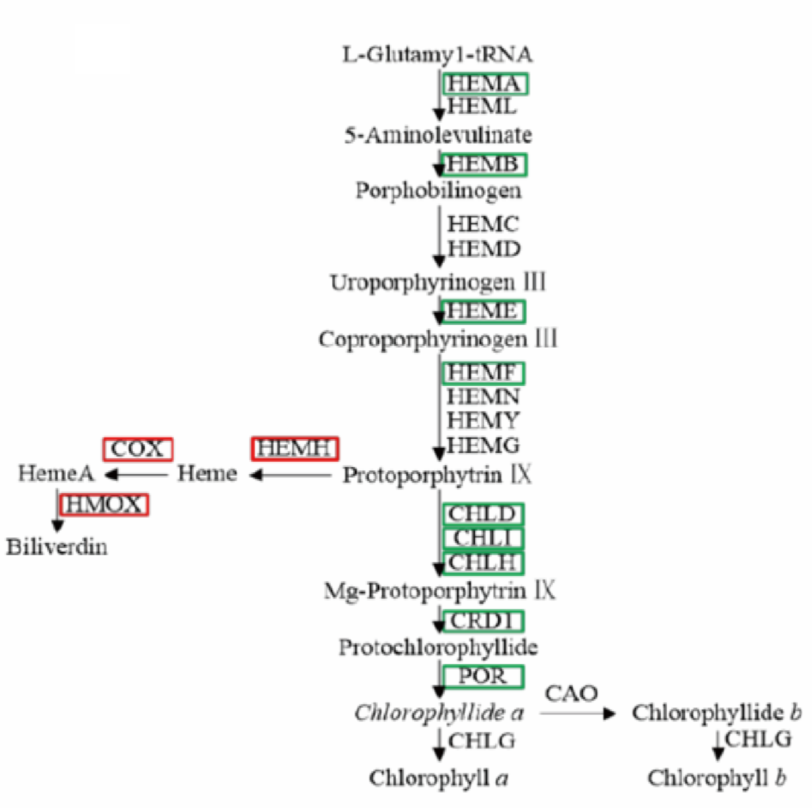

Figure S3. chlorophyll metabolic pathway of common wheat (Green indicates down-regulated expression and red indicates up-regulated expression). RNA-seq was done in the flag leaf of Common wheat variety Jinmai 39 and leave samples were collected at 0 d, 26 d and 30 d after flowering (unpublished data)

Table S1 Quality and Mapping reads of RNA-Seq

Sample	Raw Reads_count	Clean Reads_count	Q30 (%)	GC (%)	Total reads	Total mapped	Uniquely mapped	Multiple mapped
M1	91547686	90012704	94.67	53.42	90012704	84523691(93.9%)	78373864(87.07%)	6149827(6.83%)
M2	89131316	87706630	94.57	53.07	87706630	77898047(88.82%)	68579815(78.19%)	9318232(10.62%)
M3	81887406	80316034	94.84	53.63	80316034	76142421(94.8%)	70661264(87.98%)	5481157(6.82%)
M4	111664866	110585930	94.53	54.24	110585930	103756753(93.82%)	97320367(88.0%)	6436386(5.82%)
M5	90985480	88694184	94.85	50.79	88694184	81847536(92.28%)	76924741(86.73%)	4922795(5.55%)
M6	84213886	83136932	95	51.84	83136932	77722570(93.49%)	73162384(88.0%)	4560186(5.49%)
W1	85454918	83822848	94.74	53.75	83822848	79645106(95.02%)	73911660(88.18%)	5733446(6.84%)
W2	86248568	84807488	95.11	53.29	84807488	79414453(93.64%)	73418187(86.57%)	5996266(7.07%)
W3	90636228	89005610	94.67	53.54	89005610	82107718(92.25%)	74980439(84.24%)	7127279(8.01%)
W4	85008150	84155078	94.97	54.58	84155078	79602497(94.59%)	73609274(87.47%)	5993223(7.12%)
W5	92551164	91625676	95.1	54.38	91625676	86616629(94.53%)	79597507(86.87%)	7019122(7.66%)
W6	84262204	83292476	94.57	54.88	83292476	78440381(94.17%)	72709815(87.29%)	5730566(6.88%)

Table S2 List of Chlorophyll-related DEGs identified in WT and *GSm*

Gene_id	MS1	MS2	WS1	WS2	Ko name
TraesCS1A02G057200	40.71	4.04	45.97	51.37	hema
TraesCS1A02G173100	48.46	19.61	56.63	34.23	hema
TraesCS1B02G075200	33.76	3.97	41.11	52.03	hema
TraesCS1B02G191200	111.40	44.28	120.50	127.11	hema
TraesCS1D02G058300	31.38	3.85	33.71	39.24	hema
TraesCS1D02G165600	76.10	27.37	82.64	65.11	hema
TraesCS6B02G136500	0.33	0.35	0.31	1.08	hemb
TraesCS4B02G302400	7.37	5.84	9.87	10.78	hemd
TraesCS4B02G168300	38.55	29.50	37.00	27.55	heme
TraesCS2A02G426900	27.95	26.98	33.64	59.12	hemf
TraesCS2B02G447300	18.21	19.32	25.19	55.18	hemf
TraesCS2D02G425000	32.57	21.08	38.13	77.17	hemf
TraesCS6A02G213400	4.76	2.00	5.98	3.41	hemf
TraesCS6B02G243500	10.35	2.36	9.64	15.16	hemf
TraesCS6D02G196100	6.65	1.50	7.71	9.63	hemf
TraesCS7B02G234200	1.68	0.16	1.94	2.31	hemf
TraesCS2A02G347900	1.02	4.29	1.25	2.73	hemy
TraesCS2D02G346200	2.01	5.63	1.98	2.34	hemy
TraesCS5A02G045700	9.43	14.99	14.07	27.88	hemy
TraesCS5B02G049800	5.69	9.80	9.28	18.14	hemy
TraesCS5D02G055700	10.66	13.30	17.01	25.37	hemy
TraesCS2A02G134000	35.67	6.21	40.43	73.49	chlh
TraesCS2B02G157600	36.56	12.67	43.00	76.70	chlh
TraesCS2D02G136200	27.14	8.65	35.64	55.62	chlh
TraesCS5A02G466100	27.12	19.05	32.93	39.05	chld
TraesCS5B02G477800	5.92	4.16	7.47	10.49	chld
TraesCS5D02G478900	14.77	9.81	17.37	27.07	chld
TraesCS7A02G480700	68.32	51.07	67.04	116.59	chll
TraesCS7B02G382800	68.35	47.55	67.39	101.85	chll
TraesCS7D02G467500	79.41	60.19	73.39	133.27	chll
TraesCS4A02G420600	24.42	18.22	46.15	46.22	chlm

TraesCS7A02G068500	39.30	26.21	62.58	56.90	chlm
TraesCS7D02G062900	28.20	21.34	50.20	61.69	chlm
TraesCS3A02G191700	337.91	87.87	357.79	574.65	acsf
TraesCS3B02G219700	217.15	58.59	220.98	404.89	acsf
TraesCS3D02G194300	222.23	49.75	242.81	456.82	acsf
TraesCS4A02G140700	12.36	5.97	19.03	14.12	dvr
TraesCS6A02G403800	20.23	13.06	26.37	36.69	dvr
TraesCS6B02G447700	36.88	19.33	44.41	42.20	dvr
TraesCS6D02G387300	15.51	8.47	23.39	23.43	dvr
TraesCS1A02G171000	85.11	45.41	126.29	163.23	por
TraesCS1B02G186300	183.58	68.23	255.10	300.11	por
TraesCS1D02G168700	84.84	43.47	138.42	181.23	por
TraesCS2A02G590600	0.59	0.01	1.14	1.95	por
TraesCS2B02G593000	0.43	0.00	0.40	0.22	por
TraesCS2D02G563600	4.82	0.00	2.95	16.41	por
TraesCS3A02G506200	27.62	6.85	34.56	78.10	cao
TraesCS3B02G574300	23.59	4.78	29.78	68.41	cao
TraesCS3D02G514100	22.82	6.56	31.27	89.61	cao
TraesCS1A02G224700	29.46	41.43	45.20	65.68	chlg
TraesCS1B02G237700	42.88	58.52	59.70	75.55	chlg
TraesCS1D02G226100	23.11	37.38	35.39	54.62	chlg
TraesCS4A02G411000	14.14	48.78	11.93	14.18	pao /acd1
TraesCS4B02G311100	33.09	67.99	24.75	27.21	pao /acd2
TraesCS4D02G309000	29.99	71.34	28.46	26.79	pao /acd3
TraesCS3A02G151900	25.35	27.56	38.21	39.04	NOL, NYC1
TraesCS3B02G179100	28.93	32.65	44.29	38.29	NOL, NYC2
TraesCS3D02G159800	53.94	74.43	67.15	66.35	NOL, NYC3
TraesCS4A02G299400	5.63	23.45	7.22	11.53	NOL, NYC4
TraesCS4D02G012100	6.08	35.91	8.09	12.21	NOL, NYC5
TraesCS6B02G357800	6.07	34.33	6.95	10.09	NOL, NYC6

Table S3 List of carotenoid synthesis related DEGs identified in WT and *GSm*

Gene_id	MS1	MS2	WS1	WS2	KO_name
TraesCS5A02G033700	19.23	12.73	32.54	32.65	Z-ISO
TraesCS1A02G071700	11.39	8.30	12.37	13.89	crtISO
TraesCS1D02G074300	4.81	3.53	4.73	4.61	crtISO
TraesCS3A02G208800	7.43	2.93	10.53	14.57	crte
TraesCS3B02G239100	6.99	2.20	8.24	14.97	crte
TraesCS3D02G211700	10.63	4.29	16.00	18.07	crte
TraesCS6A02G171200	21.43	6.50	21.42	23.84	CrtL-b
TraesCS6D02G377900	44.12	27.40	78.71	36.11	LUT5
TraesCS1D02G210200	35.59	32.96	46.54	83.12	LUT1
TraesCS2A02G280500	36.85	14.74	47.87	22.84	VDE
TraesCS2B02G297800	47.46	23.58	56.30	35.82	VDE
TraesCS2D02G279300	35.59	19.10	44.22	28.01	VDE

Table S4 List of photosynthesis related DEGs identified in WT and *GSm*

Gene_id	MS1	MS2	WS1	WS2	KO_name
TraesCS7A02G227100	1293.96	102.48	1466.26	2119.12	LHCA1
TraesCS7B02G192500	924.69	81.95	1121.82	1661.67	LHCA1
TraesCS7D02G227300	1082.61	76.25	1318.31	1571.81	LHCA1
TraesCS2A02G187200	1794.38	183.34	2213.54	2857.69	LHCA2
TraesCS2B02G220100	1676.32	136.99	1952.99	2647.84	LHCA2
TraesCS2D02G200700	1551.23	81.63	1862.88	1929.55	LHCA2
TraesCS5A02G213000	116.21	63.55	169.86	158.73	LHCA2
TraesCS5B02G210800	72.06	21.76	87.57	125.32	LHCA2
TraesCS5D02G219100	73.87	36.70	112.82	134.78	LHCA2
TraesCS6A02G159800	620.83	65.88	809.40	1225.28	LHCA3
TraesCS6B02G191500	744.79	78.10	868.94	1267.66	LHCA3
TraesCS6D02G152700	868.93	75.77	1039.59	1538.34	LHCA3
TraesCS5A02G229300	546.03	36.40	713.22	1544.59	LHCA4
TraesCS5B02G227900	708.83	51.74	914.67	1624.98	LHCA4
TraesCS5D02G238300	779.54	62.42	1029.45	1904.87	LHCA4

TraesCS6A02G319700	84.17	25.14	117.50	84.59	LHCA5
TraesCS6B02G350500	74.65	29.21	90.42	72.77	LHCA5
TraesCS6D02G299200	104.33	35.00	128.13	118.17	LHCA5
TraesCS1A02G306600	2.01	0.10	1.98	19.53	LHCB1
TraesCS1A02G403300	461.23	10.83	608.68	1876.75	LHCB1
TraesCS1A02G403800	4.41	0.12	6.31	12.71	LHCB1
TraesCS1B02G317500	4.90	0.02	5.12	30.62	LHCB1
TraesCS1B02G388200	30.66	0.43	26.31	77.18	LHCB1
TraesCS1B02G388300	1.30	0.03	0.50	5.83	LHCB1
TraesCS1B02G388400	7.48	0.00	5.98	24.43	LHCB1
TraesCS1B02G388500	1.02	0.00	0.40	2.15	LHCB1
TraesCS1B02G432700	738.15	24.39	1122.95	2800.04	LHCB1
TraesCS1B02G433300	9.92	0.13	14.32	37.81	LHCB1
TraesCS1D02G306200	1.86	0.01	2.48	14.87	LHCB1
TraesCS1D02G374900	1.35	0.06	1.29	20.36	LHCB1
TraesCS1D02G375000	12.18	0.08	11.76	30.72	LHCB1
TraesCS1D02G375100	19.88	1.01	25.48	125.72	LHCB1
TraesCS1D02G411300	564.89	17.00	733.76	1621.80	LHCB1
TraesCS1D02G411600	4.06	0.01	7.46	19.03	LHCB1
TraesCS5A02G350600	921.71	33.28	799.98	1919.83	LHCB1
TraesCS5A02G454200	22.41	0.04	16.12	28.83	LHCB1
TraesCS5A02G454300	8.39	0.14	6.19	10.40	LHCB1
TraesCS5B02G353200	1375.10	45.20	1711.63	2644.61	LHCB1
TraesCS5B02G462800	0.37	0.00	0.31	0.74	LHCB1
TraesCS5B02G463000	7.89	0.00	5.85	27.17	LHCB1
TraesCS5B02G463100	68.33	0.03	65.24	231.55	LHCB1
TraesCS5D02G357600	831.62	17.20	718.30	1496.39	LHCB1
TraesCS5D02G464700	59.75	0.17	63.39	228.70	LHCB1
TraesCS5D02G464800	19.99	0.01	31.40	69.61	LHCB1
TraesCS5D02G464900	3.50	0.00	2.76	6.34	LHCB1
TraesCS6A02G093700	0.16	0.00	0.16	0.20	LHCB1
TraesCS6A02G094200	17.44	0.06	24.95	61.27	LHCB1
TraesCS6A02G094300	19.10	0.05	23.66	38.51	LHCB1

TraesCS6A02G094400	0.21	0.00	0.39	0.15	LHCB1
TraesCS6A02G094600	4.13	0.00	6.45	12.11	LHCB1
TraesCS6B02G122600	1.29	0.03	2.59	3.73	LHCB1
TraesCS6D02G088800	0.82	0.00	0.68	0.55	LHCB1
TraesCS7A02G238400	3.78	0.18	3.29	17.58	LHCB1
TraesCS7A02G276400	905.93	3.87	1012.44	790.79	LHCB1
TraesCS7B02G103300	0.13	0.00	0.21	0.65	LHCB1
TraesCS7B02G103400	5.23	0.26	6.83	29.12	LHCB1
TraesCS7D02G276300	482.41	3.67	603.91	707.16	LHCB1
TraesCSU02G086800	5.79	0.00	11.94	10.92	LHCB1
TraesCSU02G167700	45.66	0.53	60.24	194.25	LHCB1
TraesCSU02G250000	1.88	0.02	2.79	8.32	LHCB1
TraesCS5A02G322500	711.02	10.33	752.13	750.21	LHCB2
TraesCS5B02G322900	154.15	2.44	175.63	184.05	LHCB2
TraesCS5D02G329200	1049.96	9.03	1278.11	993.71	LHCB2
TraesCS1B02G451100	317.21	14.17	347.86	713.50	LHCB3
TraesCS1B02G451200	102.70	3.30	54.90	99.91	LHCB3
TraesCS1D02G428200	396.37	21.62	396.68	907.13	LHCB3
TraesCS2A02G204800	198.63	8.15	220.35	258.71	LHCB3
TraesCS2A02G206200	1133.81	60.96	1465.33	1769.44	LHCB4
TraesCS2B02G233400	762.90	38.13	977.92	1101.55	LHCB4
TraesCS2D02G209900	956.51	51.31	1228.85	1678.34	LHCB4
TraesCS4A02G226900	1238.10	48.27	1463.41	1463.39	LHCB5
TraesCS4B02G089500	1216.75	45.08	1501.33	1572.97	LHCB5
TraesCS4D02G086400	1066.18	57.81	1355.81	1500.13	LHCB5
TraesCS2A02G342600	478.70	21.38	571.60	570.64	LHCB6
TraesCS2B02G340300	535.07	32.77	624.41	718.55	LHCB6
TraesCS2D02G320900	450.35	27.11	497.71	604.52	LHCB6
TraesCS5B02G175000	16.88	15.66	22.87	17.72	LHCB7

Table S5 List of enzyme activity-related DEGs identified in WT and *GSm*

Group	Gene_id	TPM				KO_name
		MS1	MS2	WS1	WS2	
Superoxide dismutase	*TraesCS2A02G121200*	173.59	364.35	94.25	111.28	SOD1
	TraesCS2D02G123300	153.27	251.15	105.86	102.15	SOD1
	TraesCS4A02G065800	48.08	32.84	52.79	64.51	SOD1
	TraesCS4B02G243200	71.01	76.14	93.24	118.59	SOD1
	TraesCS4D02G242800	31.74	21.51	30.23	41.25	SOD1
	TraesCS7A02G292100	48.82	34.37	58.62	83.73	SOD1
	TraesCS7B02G197300	44.26	55.62	52.10	84.77	SOD1
	TraesCS7D02G290700	61.30	54.88	67.05	94.29	SOD1
	TraesCS2A02G537100	46.14	38.93	43.25	24.10	SOD2
	TraesCS2B02G567600	9.00	13.25	5.47	9.10	SOD2
	TraesCS2D02G538300	60.43	73.14	62.83	39.19	SOD2
	TraesCS4A02G390300	3.97	14.72	6.86	11.01	SOD2
	TraesCS4A02G434000	4.50	7.76	5.56	9.10	SOD2
	TraesCS7A02G048600	4.21	6.43	4.95	6.78	SOD2
	TraesCS7A02G090400	9.92	18.11	19.92	24.73	SOD2
	TraesCS7D02G043000	10.96	31.84	13.93	26.17	SOD2
	TraesCS7D02G086400	12.22	28.12	21.59	31.61	SOD2
Catalase	*TraesCS4B02G325800*	491.64	359.75	527.76	396.66	katE, CAT, catB, srpA
	TraesCS4D02G322700	348.77	260.92	381.72	283.34	katE, CAT, catB, srpA
	TraesCS5A02G498000	272.65	232.45	344.57	244.90	katE, CAT, catB, srpA
	TraesCS6A02G041700	3.56	234.12	3.87	6.69	katE, CAT, catB, srpA
	TraesCS6B02G056800	0.11	14.20	0.28	0.59	katE, CAT, catB, srpA
	TraesCS6B02G330700	14.36	25.85	9.64	12.60	------
	TraesCS6D02G048300	4.68	115.62	4.92	13.00	katE, CAT, catB, srpA
	TraesCS7A02G549800	11.58	116.59	10.23	46.10	katE, CAT, catB, srpA
	TraesCS7A02G549900	0.74	6.55	0.38	3.76	katE, CAT, catB, srpA
	TraesCS7B02G473400	3.58	16.07	2.69	5.95	katE, CAT, catB, srpA
	TraesCSU02G105300	16.78	98.39	13.81	36.82	katE, CAT, catB, srpA

	TraesCS2A02G582000	7.05	1.32	0.26	0.88	------
	TraesCS2A02G582100	12.46	3.73	15.89	14.03	gpx, btuE, bsaA
	TraesCS2A02G582200	12.58	2.55	12.11	7.07	gpx, btuE, bsaA
	TraesCS2B02G429100	19.81	46.42	12.52	16.89	gpx, btuE, bsaA
	TraesCS2B02G604800	31.97	9.73	37.87	13.61	gpx, btuE, bsaA
	TraesCS2B02G604900	26.81	7.21	29.72	8.53	gpx, btuE, bsaA
	TraesCS2B02G605000	5.73	2.06	0.29	1.27	------
	TraesCS2D02G407700	21.38	51.72	18.38	15.81	gpx, btuE, bsaA
	TraesCS2D02G598000	7.81	5.05	10.44	6.59	gpx, btuE, bsaA
	TraesCS2D02G598100	3.38	3.70	5.04	0.31	gpx, btuE, bsaA
	TraesCS2D02G598200	3.74	1.66	0.10	0.68	------
	TraesCS4A02G142000	40.08	90.41	31.78	58.33	gpx, btuE, bsaA
	TraesCS4A02G269600	24.23	51.41	10.10	20.96	GST, gst
	TraesCS4A02G269800	45.71	34.08	20.61	18.93	GST, gst
	TraesCS4B02G044300	48.70	85.29	18.17	36.03	GST, gst
	TraesCS4B02G044500	22.02	12.53	2.20	6.02	GST, gst
Glutathione S-transferases	*TraesCS4B02G152800*	35.98	93.22	31.91	49.00	gpx, btuE, bsaA
	TraesCS4D02G042000	41.43	49.48	7.92	21.93	GST, gst
	TraesCS4D02G162000	42.14	92.35	37.40	60.35	gpx, btuE, bsaA
	TraesCS6A02G246400	59.66	68.55	76.67	52.41	gpx, btuE, bsaA
	TraesCS6B02G278100	16.91	21.83	17.03	8.35	gpx, btuE, bsaA
	TraesCS6D02G228800	29.52	34.02	38.95	19.15	gpx, btuE, bsaA
	TraesCS7A02G150500	141.08	100.64	307.06	381.00	gpx, btuE, bsaA
	TraesCS7B02G054400	27.95	18.08	51.26	97.35	gpx, btuE, bsaA
	TraesCS7D02G152400	113.24	61.33	245.38	262.12	gpx, btuE, bsaA
	TraesCS1A02G077700	1.92	10.01	1.69	1.31	E1.11.1.7
	TraesCS1A02G108400	0.65	4.21	0.23	1.38	E1.11.1.7
	TraesCS1B02G095800	1.40	8.39	0.85	2.50	E1.11.1.7
	TraesCS1D02G096400	0.98	6.11	0.12	1.01	E1.11.1.7
	TraesCS1D02G174200	5.53	14.31	7.33	9.83	E1.11.1.7
	TraesCS2A02G263500	53.48	83.64	71.36	55.28	E1.11.1.7
	TraesCS2A02G333500	2.01	17.69	2.35	0.69	E1.11.1.7

	TraesCS2A02G571300	2.37	12.73	1.68	7.06	E1.11.1.7
	TraesCS2B02G278500	21.85	19.37	27.87	23.79	E1.11.1.7
	TraesCS2B02G346800	1.74	10.90	1.45	0.43	E1.11.1.7
	TraesCS2B02G613400	5.65	9.28	5.78	6.98	E1.11.1.7
	TraesCS2B02G614100	0.10	18.47	0.00	0.00	E1.11.1.7
	TraesCS2D02G260300	31.65	12.33	43.01	12.83	E1.11.1.7
	TraesCS2D02G327700	1.59	5.17	2.09	0.95	E1.11.1.7
	TraesCS2D02G583000	4.25	25.55	2.41	13.40	E1.11.1.7
	TraesCS2D02G583200	0.50	1.75	0.01	0.60	E1.11.1.7
	TraesCS3A02G183500	25.23	26.10	35.76	29.28	------
	TraesCS3A02G185900	6.04	110.10	4.84	0.86	E1.11.1.7
	TraesCS3A02G297100	0.11	0.96	0.00	0.03	E1.11.1.7
	TraesCS3A02G297200	0.22	18.85	0.19	0.29	E1.11.1.7
	TraesCS3A02G325100	1.72	13.47	1.99	0.55	E1.11.1.7
	TraesCS3B02G213200	1.03	5.60	1.69	2.66	------
	TraesCS3B02G215500	4.59	50.73	8.12	0.85	E1.11.1.7
	TraesCS3B02G354000	0.63	6.13	0.50	0.18	E1.11.1.7
	TraesCS3B02G578600	0.03	5.08	0.00	0.01	E1.11.1.7
	TraesCS3D02G187600	13.97	12.72	16.18	15.23	------
Peroxidase	*TraesCS3D02G189900*	2.31	86.67	1.35	0.16	E1.11.1.7
	TraesCS3D02G305300	0.06	2.23	0.11	0.11	E1.11.1.7
	TraesCS3D02G318500	1.59	12.68	0.89	0.44	E1.11.1.7
	TraesCS3D02G518200	0.23	5.43	0.00	0.13	E1.11.1.7
	TraesCS4A02G196300	0.00	6.59	0.00	0.01	E1.11.1.7
	TraesCS4A02G196400	0.03	6.63	0.00	0.00	E1.11.1.7
	TraesCS4D02G342600	0.57	5.34	1.45	0.08	E1.11.1.7
	TraesCS5A02G249200	5.26	6.07	6.32	4.63	E1.11.1.7
	TraesCS5A02G288200	0.57	14.80	0.73	0.55	E1.11.1.7
	TraesCS5A02G400500	0.75	15.09	0.03	0.04	E1.11.1.7
	TraesCS5B02G246900	8.54	10.67	4.57	3.76	E1.11.1.7
	TraesCS5B02G405300	0.13	17.63	0.00	0.10	E1.11.1.7

	TraesCS5D02G256400	5.95	5.49	6.12	4.33	E1.11.1.7
	TraesCS5D02G410500	0.16	12.83	0.04	0.14	E1.11.1.7
	TraesCS6A02G118300	5.50	6.22	3.03	2.77	E1.11.1.7
	TraesCS6B02G146500	2.78	10.21	2.10	1.53	E1.11.1.7
	TraesCS6D02G108400	5.10	20.40	4.93	3.45	E1.11.1.7
	TraesCS7A02G250500	14.72	14.48	25.03	38.57	E1.11.1.11
	TraesCS7A02G319100	0.21	8.52	0.07	0.03	E1.11.1.7
	TraesCS7A02G339600	1.45	16.43	0.26	0.64	E1.11.1.7
	TraesCS7A02G428200	4.46	9.82	6.10	1.76	E1.11.1.7
	TraesCS7B02G099800	2.38	10.57	1.40	1.91	------
	TraesCS7B02G140600	15.08	20.70	30.30	23.55	E1.11.1.11
	TraesCS7B02G328400	2.05	20.18	2.28	1.43	E1.11.1.7
	TraesCS7D02G212900	2.65	13.07	1.87	2.38	E1.11.1.7
	TraesCS7D02G249200	17.94	18.19	32.03	40.81	E1.11.1.11
	TraesCS7D02G347300	0.69	3.80	0.13	0.58	E1.11.1.7
	TraesCS7D02G420500	2.79	75.08	4.11	2.20	E1.11.1.7

Table S6 List of cytokinin and gibberellin-related DEGs identified in WT and *GSm*

Group	Gene_id	TPM			
		MS1	MS2	WS1	WS2
CK	*TraesCS1B02G137600*	1.40	0.12	2.07	0.31
	TraesCS1B02G176000	0.29	0.95	0.14	0.58
	TraesCS1B02G323900	0.00	0.06	0.00	0.00
	TraesCS2A02G265700	2.72	5.16	3.49	2.61
	TraesCS2B02G150300	0.52	1.25	0.15	0.39
	TraesCS2D02G407200	0.15	2.08	0.64	0.79
	TraesCS2D02G407300	4.73	9.19	6.52	8.99
	TraesCS3A02G109500	0.00	0.04	0.00	0.00
	TraesCS3A02G311100	0.30	0.08	0.00	0.05
	TraesCS3B02G128700	0.00	0.02	0.00	0.02
	TraesCS3B02G344600	1.29	1.79	0.99	0.63

	TraesCS3D02G127100	1.41	4.64	0.38	1.09
	TraesCS3D02G263000	0.71	0.73	0.44	0.08
	TraesCS3D02G310200	1.50	0.42	0.25	0.25
	TraesCS4A02G345200	0.87	1.25	1.29	1.59
	TraesCS5A02G444500	1.46	0.47	2.20	0.14
	TraesCS5B02G528800	1.26	0.74	1.01	0.79
	TraesCS7A02G536900	3.34	2.00	7.04	17.86
	TraesCS7A02G560300	0.53	0.74	0.54	0.96
	TraesCS7B02G075300	0.17	0.01	0.16	0.02
	TraesCS7B02G455000	2.93	2.99	4.48	7.47
	TraesCS7D02G171100	0.21	0.02	0.09	0.07
	TraesCS7D02G171200	0.23	0.02	0.18	0.09
	TraesCS7D02G190600	2.07	23.94	6.94	0.57
GA	*TraesCS2A02G027800*	0.02	0.00	0.02	0.19
	TraesCS2A02G425500	2.28	13.74	4.03	11.05
	TraesCS2B02G396000	0.04	0.15	0.03	0.00
	TraesCS2B02G445300	15.62	6.11	29.58	19.02
	TraesCS2B02G445500	0.01	0.01	0.01	0.20
	TraesCS2B02G445600	0.01	0.09	0.02	0.31
	TraesCS2B02G445900	1.33	0.01	2.31	0.39
	TraesCS2D02G423500	72.63	0.93	113.47	56.12
	TraesCS3A02G086100	0.10	0.00	0.04	0.00
	TraesCS3A02G294000	0.17	2.69	0.11	0.01
	TraesCS3B02G328700	0.07	2.64	0.04	0.00
	TraesCS3D02G124500	0.01	0.17	0.03	0.00
	TraesCS3D02G293800	0.02	1.04	0.02	0.00
	TraesCS4A02G460100	4.13	0.11	6.09	3.84
	TraesCS4B02G376200	1.30	6.22	0.65	0.87
	TraesCS5A02G064200	1.12	0.30	1.98	1.99
	TraesCS5B02G068500	0.46	0.19	0.36	1.13
	TraesCS7A02G029600	0.74	0.22	1.01	0.16

TraesCS7A02G362300	0.78	1.12	2.35	12.60
TraesCS7A02G552700	0.19	0.08	0.17	0.03
TraesCS7B02G476400	0.15	0.05	0.11	0.04
TraesCS7D02G026000	4.70	0.20	7.71	4.72
TraesCS7D02G539200	0.60	0.70	1.07	0.91
TraesCSU02G008700	0.03	0.01	0.06	0.48
TraesCSU02G008900	0.01	0.02	0.04	0.30
TraesCSU02G009100	0.71	0.99	0.14	0.61

Table S7 Primers for quantitative real-time polymerase chain reaction

Gene_id	Forward primer	Reverse primer
TraesCS1D02G232700	CCCAGGGTCGTGGATGACTT	GCCTGGTACTTGTCCCGGTAGA
TraesCS2D02G163300	CTTCTACCTTGTTCAGCAATGGC	ACTTCTTCTTGCGCTTGGTCAC
TraesCS3D02G017700	TCACCAAGATGGACAAGGCG	ATGCGTGGCAGGAGAAGTGAC
TraesCS3D02G223300	CGAAAAGGAGGCAGTGAACGT	ACTGGAAAACAACGAGTACGCAC
TraesCS5B02G115300	AGGCCACGCTCAACCTCAATG	GCCGTCGCTGGTGTACTTTCC
TraesCS7A02G319100	CTGCTTCGTACAGGGATGCG	CGGTCAGCGTGTCGGTGT
